# How to build machine learning models able to extrapolate from standard to modified peptides

**DOI:** 10.1186/s13321-025-01115-z

**Published:** 2025-11-27

**Authors:** Raúl Fernández-Díaz, Rodrigo Ochoa, Thanh Lam Hoang, Vanessa Lopez, Denis C. Shields

**Affiliations:** 1 IBM Research, Dublin, Ireland; 2 Novo Nordisk A/S, Måløv, Denmark; 3 Conway Institute for Biomolecular and Biomedical Research, University College Dublin, Dublin, Ireland; 4 School of Medicine, University College Dublin, Dublin, Ireland; 5 The Research Ireland Center for Research Training on Genomics Data Science, Galway, Ireland

## Abstract

Bioactive peptides are an important class of natural products with great functional versatility. Chemical modifications can improve their pharmacology, yet their structural diversity presents unique challenges for computational modeling. Furthermore, data for standard peptides (composed of the 20 canonical amino acids) is more abundant than for modified ones. Thus, we set out to identify whether predictive models fitted to standard data are reliable when applied to modified peptides. To do this, we first considered two critical aspects of the modeling problem, namely, choice of similarity function for guiding dataset partitioning and choice of molecular representation. Similarity-based dataset partitioning is an evaluation technique that divides the dataset into train and test subsets, such that the molecules in the test set are different from those used to fit the model.

**Graphical Abstract:**

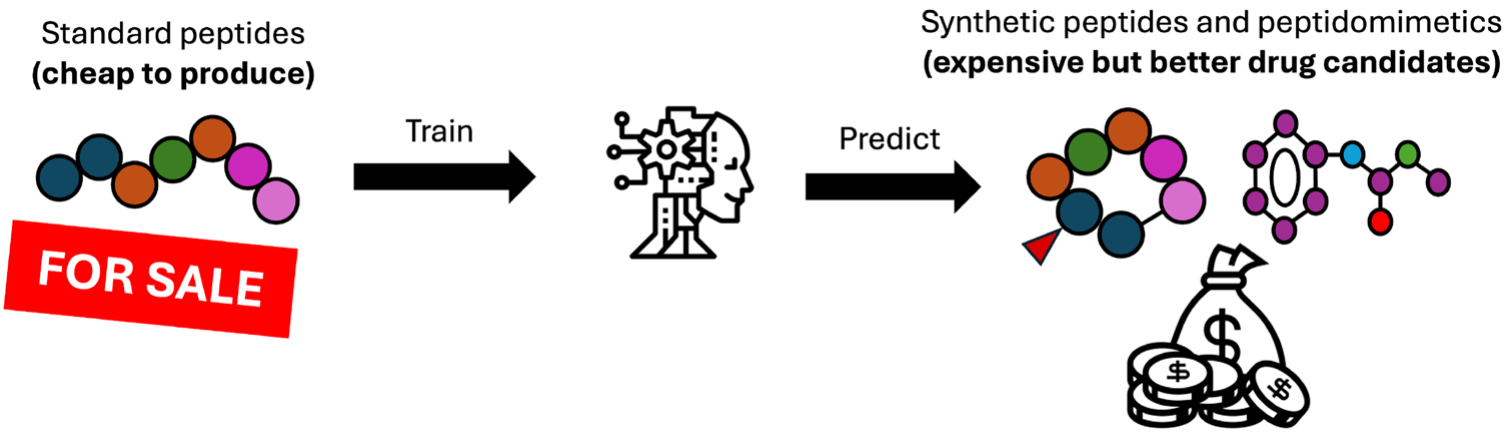

## Scientific contribution

We demonstrate, across four peptide function prediction tasks, that chemical fingerprint-based similarity measures outperform traditional sequence alignment-based metrics for partitioning standard peptide datasets, challenging conventional practice. We have also found that the choice of representation does not have a substantial impact in the standard to standard interpolation modelling scenario, but for modified to modified interpolation chemical language models are the best option. Despite a 50% drop in performance, chemical fingerprints and a chemical language model (ChemBERTa-2) are the best choices in the more challenging extrapolation scenario (standard to modified). All code and data necessary for reproducing the experiments are available in Github (https://github.com/IBM/PeptideGeneralizationBenchmarks).

## Introduction

Peptides are polymers of amino acids that play important roles in many biological processes, acting as signaling molecules in metabolic and immunological pathways for a great diversity of organisms. They occupy an interesting position in biochemistry, between proteins and small organic compounds. Peptide engineering methods [1–3] allow for the design of selective binders which makes them an increasingly important drug modality for pharmaceutical development. Robust computational methods for predicting peptide pharmacological properties like their ability to penetrate cells or their bioactivity, will further accelerate their incorporation into drug development pipelines [4, 5].

However, often standard peptides (those comprised of the 20 canonical amino acids) are not effective drugs due to suboptimal pharmacological properties like solubility, absorption, or persistence. Chemical modifications of these peptides, including alterations to side-chains, inclusion of non-canonical amino acids, or cyclizations, can greatly improve their pharmacology. From a modelling perspective, the increased diversity of modified peptides introduces multiple challenges [4, 6].

These challenges are further exacerbated by the scarcity of publicly annotated data [5] and the inconsistent annotation of chemical modifications in most available repositories, where custom notations [7–9] are used instead of structured formats like SMILES (Simplified Molecular Input Line Entry System) [10], HELM (Hierarchical Editing Language for Macromolecules) [11], or BILN (Boehringer-Ingelheim Line Notation) [12]. Moreover, obtaining experimental data for modified peptides is generally more costly due to their more complex synthetic strategies [13]. Thus, there is a need for modeling strategies that leverage the more abundant standard peptide data to build models that can effectively extrapolate to modified peptides, bypassing these limitations.

Our objective in this study is to discern whether ML models are able to extrapolate from one peptide class (standard) to another (modified). In other words, can we train models in standard peptide data that make reliable predictions for modified peptides?

The baseline for this extrapolation scenario are the interpolation scenarios: standard to standard and modified to modified. These scenarios represent model performance when predicting new molecules belonging to the same molecular class. In order to obtain reliable baselines that measure the ability of models to generalize to new molecules belonging to the same molecular class, it is necessary to partition the datasets into training and testing subsets such that they contain molecules as different from each other as possible.

There are multiple strategies available for dataset partitioning for biosequences [14], molecules [15–20], or both[21, 22]; however, there is no prior literature describing how to perform this type of dataset partitioning (generally known as, similarity-based Out-Of-Distribution, OOD, partitioning) for modified peptides. This is the first question that we have addressed in this study: we evaluated different similarity-based OOD configurations to find those most appropriate for peptide datasets.

Finally, the way in which we represent the peptides, i.e., the way in which we translate the molecular graphs into a mathematical expression that the model is able to interpret, has been shown to have a very important role in model performance. [23, 24] Traditional techniques for peptide representation are focused on standard peptides and rely on sequence-based, physico-chemical, or evolutionary properties [5]. These properties do not extend to the sheer diversity of possible non-canonical monomers. Chemical representation methods [25, 26], on the other hand, by interpreting peptides as molecular graphs are more versatile and can naturally consider most chemical modifications [23], but their use for peptide representation in the context of predictive modeling [5] is not as extensively described.

Modern representation techniques, both for biosequences and molecules, rely mostly on self-supervised pre-trained deep learning models or foundation models (FM). They can be divided by architecture into i) transformers-based, often named Language Models (LMs) or ii) graph neural network-based, or GNN-based for short. They can also be divided by their pre-training data into a) general chemistry models (trained on vast chemical libraries including both small drug-like compounds and larger polymers and macromolecules) [27, 28], b) peptide-specific models (trained on specialized libraries of peptide molecules [29, 30], or c) protein-specific models (trained on vast libraries of protein sequences) [31, 32]. The second main question that we have addressed in this study is what representation methods are the best suited for peptide modelling in interpolation and/or extrapolation scenarios.

**Fig. 1:**
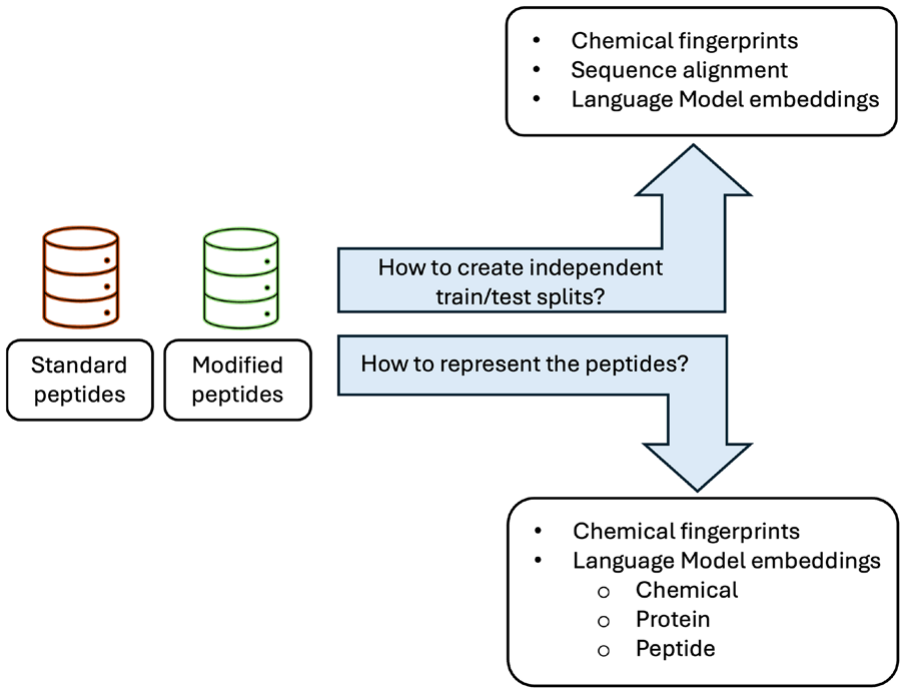
Visual summary of main research questions

In summary, the two main technical questions that we have addressed in this study are: 1) How can we generate good OOD train/test splits for peptide data (standard or modified) and 2) What are the best ways to represent peptides for ML to learn in a) interpolation and b) extrapolation scenarios. Figure 1 provides a visual schematic of these research questions.

We have prepared 8 datasets covering 4 biophysical property predictions tasks where for each task we have two datasets, one that only contains standard peptides and the other only modified ones. We have found that chemical fingerprints performed better than sequence-based methods both in defining peptide similarity for dataset partitioning and on training predictive models for modified to modified interpolation and standard to modified extrapolation; and we found no significant difference between sequence-based approaches and chemical fingerprints for standard to standard interpolation. We use this results to provide insights and guidance regarding the practicality of learning from standard peptide datasets to make functional predictions for modified peptides.

### Computational methodology

#### Data collection

We collected 8 datasets from prior publications (see Table 1). Two datasets focused on protein-peptide binding affinity, of which the first contained a mixture of standard and modified peptides and the second, solely modified peptides [30]. The first dataset was filtered to remove all modified peptides.

Two additional datasets concerned cell membrane penetration. One contained standard peptides and was structured as a classification task, while the other one only contained modified peptides and was conceived as a regression task [30]. For consistency, we redefined the latter dataset into a classification task (more details in Supplementary SA).

**Table 1:** Benchmark datasets. *: Dataset has been modified in this study (More details in Supplementary SA)

Dataset	Type	Task	Size	Source
Protein-peptide binding affinity	Standard	Regression	1,002	[30]
Protein-peptide binding affinity	Modified	Regression	299	[30], *
Cell penetration	Standard	Classification	2,324	[30], *
Cell penetration	Modified	Classification	480	[30], *
Antibacterial	Standard	Classification	9,855	[24], *
Antibacterial	Modified	Classification	1,880	[33], *
Antiviral	Standard	Classification	4,754	[24], *
Antiviral	Modified	Classification	444	[33], *

The remaining four datasets concerned classification of antibacterial and antiviral peptides. For the standard datasets, a preprocessing step was performed to remove any sequence longer than 50 residues, according to the conventional definition for peptides [5]. For modified peptide datasets we gathered two datasets with only positive samples [33], and sampled negative peptides by drawing them from other bioactivities described in the same study (i.e., for the Antibacterial dataset, the negatives were drawn from the Antifungal, Antiparasitic, and Antiviral datasets; and for the Antiviral, from Antibacterial, Antifungal, and Antiparasitic), in such a way as to ensure that both positive and negative peptide have similar distributions of molecular weights. This approach follows the negative peptide sampling method introduced in Fernández-Díaz et al. [24]. Thus, these prediction tasks may be more precisely defined as the identification of antibacterial (or antiviral) peptides within the broader category of antimicrobial peptides.

### Model training and hyperparameter optimization

**Fig. 2:**
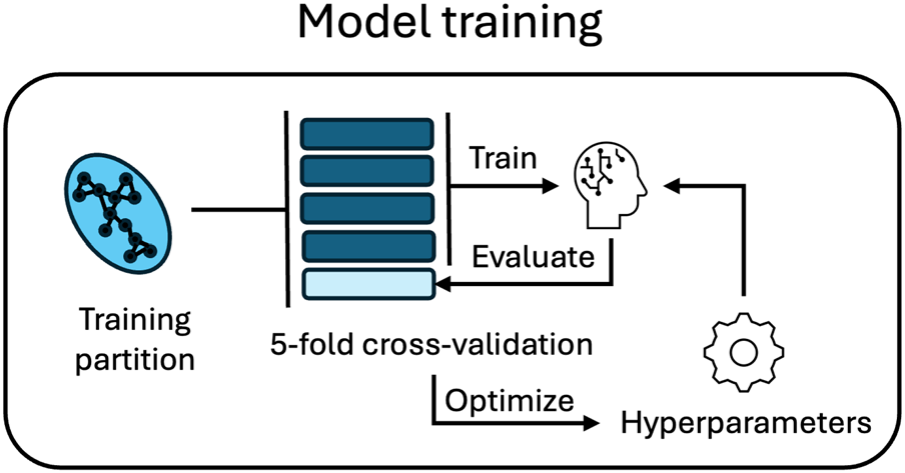
Visual representation of the model training module

Two ML algorithms were used in this study: support vector machine (SVM) and Light Gradient Boosting (LightGBM). In each experiment one of the two algorithms was used to train a predictive model. As these algorithms have multiple settings (known as hyperparameters), we performed hyperparameter optimization (HPO) in 5-fold cross-validation, for each experiment independently. HPO was performed through the Optuna implementation [34] of bayesian optimization, with default settings for 200 steps with 10 parallel threads and early stopping with a 20 step patience. Table S1 describes the hyperparameter spaces explored for each algorithm. Figure 2 provides a visualization of the hyperparameter optimization loop.

All training experiments are repeated five times with different seeds for the pseudorandom number generators to account for the randomness inherent to the stochastic process in model learning and HPO.

### Search for optimal dataset partitioning strategy

The objective of similarity-based dataset partitioning is to divide the dataset into a training and evaluation subsets, such that the evaluation subset contains molecules different from those seen in training. One of the most important variables for defining good training/evaluation partitions is how the similarity between the molecules is defined. We refer to the different strategies for measuring molecular similarity as similarity functions.

There are many different similarity functions that could be used for peptide datasets. These functions can be classified into bioinformatic, which only apply to standard peptides; and cheminformatic, which apply to both standard and modified peptides. Among the bioinformatic tools, we considered different sequence alignment tools including local (MMseqs2 with and without prefiltering) [35], and global (EMBOSS needleall implementation of the Needleman-Wunsch algorithm) [36] alignment algorithms. Among the cheminformatic tools, we considered the Jaccard similarity for two types of chemical fingerprints, the extended connectivity fingerprints (ECFP) [25] and the MinHashed Atom-Pair chiral fingerprints (MAPc) [37]. We evaluated the fingerprints with multiple diameters from 4 to 20.

We also considered new similarity functions derived from the Chemical Language Model Molformer-XL [27] (for standard and modified peptide datasets) and the Protein Language Model ESM2–8 M [32] (for standard datasets), both with euclidean similarity, as described in Fernandez-Diaz et al. [22], More details about the implementation of each similarity function can be found in Supplementary SB.

**Fig. 3:**
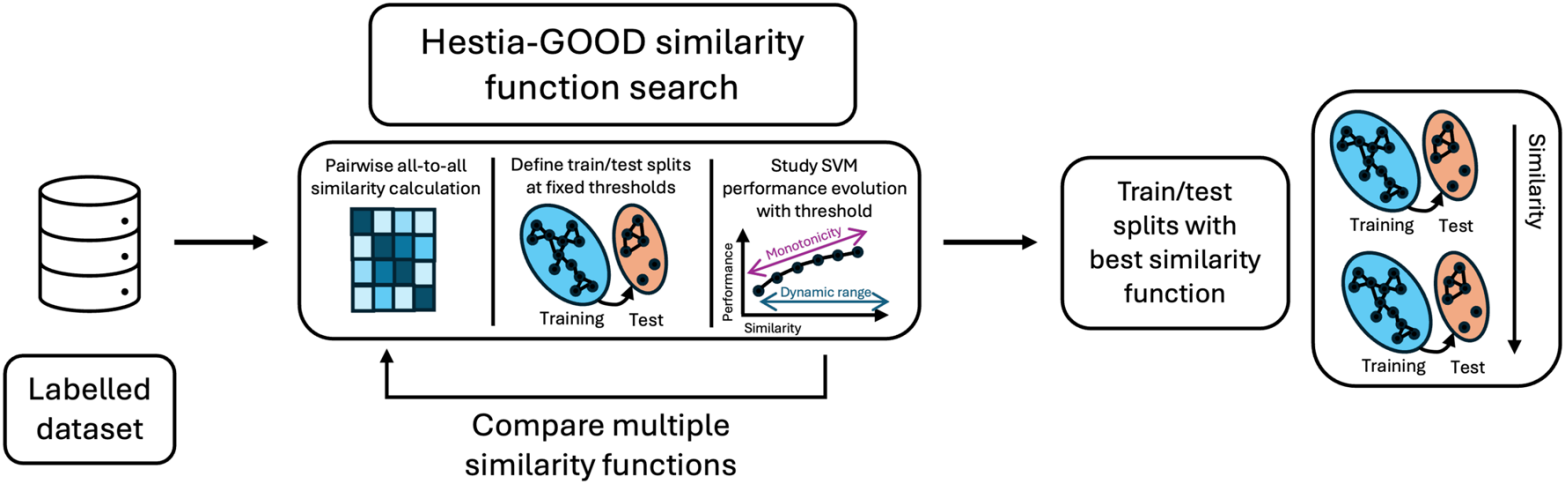
Identification of the best similarity function for peptide dataset partitioning (1–6) and evaluation of representation methods (7 and 8) using the Hestia-GOOD framework

Figure 3 illustrates the workflow used for determining the best similarity function. For all datasets and similarity functions described above: 1) the pairwise similarities between all peptides are calculated; 2) the similarities are used to define train/test splits at increasing similarity thresholds using the CCPart algorithm [22]; 3), the evolution of model performance as a function of the train-test similarity is studied to determine which similarity function provides the most relevant partitions.

A “biologically” relevant similarity function for dataset partitioning will divide the dataset into train/test partitions such that as the similarity threshold increases, the chemical spaces represented in train and test subsets grow more similar to each other, and in response the evaluated model performance also increases. To evaluate the similarity functions, we trained and tested a simple machine learning model (SVM with ECFP-16 fingerprints as features) in all viable partitions for each similarity function and the we calculated the correlation between similarity threshold and model performance. As the shape of the similarity-model performance curve (i.e., GOOD-curve) may not be linear, we considered the non-linear correlation or monotonicity through Spearman’s $\rho$ coefficient[22].

We considered a given partition as valid (and therefore included in the analysis) if the test subset contained at least 18.5% of the data (the conventional 20% with a 1.5% margin of error). The number of viable partitions obtained in each experiment is referred to as the dynamic range of the similarity function, with higher dynamic ranges corresponding to functions able to make finer-grained distinctions between similar molecules [22].

We chose the ECFP-16 chemical fingerprints for these models, as it is a simple and conventional representation that has been shown to be effective in peptide function prediction [23] and SVM as a good performing model that requires minimal computational resources. As shown in Figures S7–8, the shape of the curves remains similar regardless of the choice of model or representation technique [22].

The choice of similarity function per dataset was determined according to the following criteria:

1. All similarity functions with monotonicity lower than 0.4 were filtered out, as they do not fulfill the necessary assumption of correlation between similarity threshold and model performance.
2. All similarity functions with dynamic range inferior to the maximum dynamic range across all similarities were filtered out. This filter included an arbitrary margin $\delta$, to ensure that not only the similarity function with the largest dynamic range is considered. In this study, $\delta _0=0.2$.
3. If the number of similarity functions that passed the two previous filters was smaller than an arbitrary number *k*, then step 2 was repeated with $\delta _{i+1}=\delta _i+\epsilon$, where $\epsilon$ is the step between thresholds; until either there were *k* functions that passed the filter in Step 2; or there were no more functions available. In this study, $k=3$ and $\epsilon =0.1$.
4. The similarity function with the best monotonicity was selected from among the functions available after Step 3.

### Evaluation of representation techniques

#### Representation techniques

The representation techniques considered in this study are as follows:

1. Chemical fingerprints or Chemical FPs: i) the ECFP-16 (2,048 bits) [25] as binary fingerprints or as count fingerprints (for simplicity, the term ECFP will be reserved for the binary version) and ii) the Avalon fingerprints (2,048 bits) [26].
2. Peptide fingerprints or Peptide FPs: monomer-based PepFuNN fingerprints [38].
3. General chemical language models or CLMs (trained on small organic compounds): ChemBERTa-2 77 M MLM [28] and the publicly available version of Molformer-XL [27].
4. A peptide language model or Peptide LM (trained on peptide SMILES strings): PeptideCLM [29].
5. A pre-trained multi-view heterogeneous graph neural network or Peptide GNN (trained on explicit peptide molecular graphs): Pepland [30].
6. Protein language models or PLMs (trained on protein sequences), only for the standard peptides: ESM2 8 M, 150 M, 650 M [32], ProtBERT, and Prot-T5-XL (650 M) [31].

The learned representations were calculated using the publicly available weights in the Huggingface transformers library [39], with the exception of Pepland where we used the weights in their public repository^1^ (More information on Supplementary SC.2). To use the PLMs with modified peptides, the peptide bonds were computationally broken and the sequence was reconstructed from the existing monomers. There are two possible strategies, introducing i) the ‘X’ unknown amino acid or ii) their natural analog, as defined by the ChEMBL 35 monomer library [40]. We conducted some experiments (Table S2) that showed no clear advantage of one method over the other. We decided to choose option i).

#### Experimental designs

**Fig. 4:**
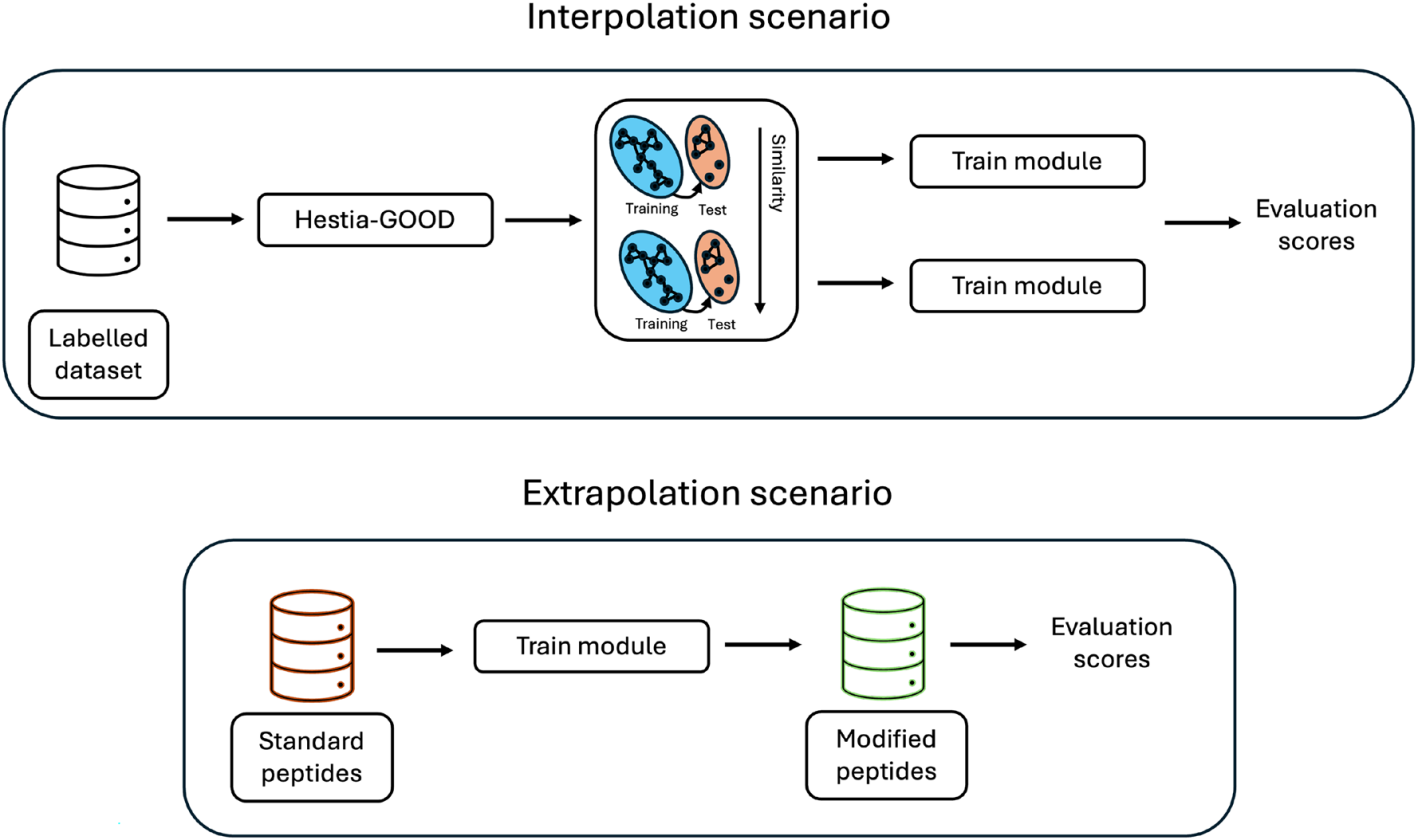
Difference between the workflows in the interpolation and extrapolation scenarios

The two ML models (SVM and LightGBM) and the different representations were evaluated under two scenarios: interpolation (standard to standard or modified to modified) and extrapolation (standard to modified). Figure 4 provides a graphical summary of the difference between both scenarios.

*Interpolation scenario*. The train/test partitions generated with the best similarity function defined in the previous subsection for each dataset were then used to evaluate different machine learning models and peptide molecular representations. For each dataset and threshold, a model was trained on the training set and evaluated against the corresponding test set.

*Extrapolation scenario*. For each of the biophysical property prediction tasks, the models were trained on the whole standard dataset and evaluated against the whole modified dataset.

#### Statistical tests

We followed recent community guidelines for the statistical analysis of machine learning model comparisons [41].

*Analysis of interpolation experiments*. For evaluating the effect of model representation across different similarity thresholds, we performed Kruskal-Wallis, as model performance cannot be assumed to be normally distributed in this scenario [22]. To compare the representation methods to each other, wherever the Kruskal-Wallis test showed significant effect, we performed pairwise one-tailed Wilcoxon rank-signed sum tests, as a *post-hoc* analysis to determine which representation methods were significantly better than the others. The Bonferroni correction for multiple testing was applied to the significance threshold [42].

*Analysis of extrapolation experiments*. For evaluating the effect of model performance, when we do not compare across multiple similarity thresholds, we performed ANOVA with *post-hoc* Tukey’s Honestly Significant Differences test (Tukey HSD).

Finally, we grouped the representations into tiers or Significant ranks according to the results from the *post-hoc* tests. First, we ranked all representations according to their average performance across all experiments; then we assigned rank 1st to the top representation. Iteratively, we checked the following representation(s), if it was not significantly different from the first representation in the current rank it received the same rank; otherwise it received the next rank. This is repeated until all representations have an associated rank.

## Results and discussion

The main results from this study are broken down into three separate subsections: 1) we report the most appropriate similarity functions for guiding peptide dataset partitioning from a collection of bio and cheminformatic tools; 2) using the datasets partitioned with the best similarity functions, we explore the best and most general representation technique for modeling standard and modified peptides separately; and 3) we determine what representations allow models trained on only standard peptides to extrapolate to modified peptides.

### Search for optimal similarity functions to guide dataset partitioning

There is no prior work systematically evaluating the optimal similarity functions to use for similarity-based dataset partitioning for peptide datasets. So, we relied on the Hestia-GOOD framework to perform this exploration, focusing on two quantitative metrics for each similarity metric: 1) dynamic range, or the difference between the minimum and maximum similarities at which viable train/test splits can be generated; and 2) monotonicity (Spearman’s $\rho$ correlation), or the dependence between model performance and similarity threshold [22].

**Table 2:** Best similarity function identified for each dataset considered. $\uparrow$: Greater is better. [a] Difference between the minimum and maximum similarity threshold at which viable train/test partitions can be generated. [b] Spearman’s $\rho$ correlation coefficient between model performance and similarity threshold

Dataset	Peptide type	Task	Similarity Type	Similarity	Dynamic	Monotonicity
					range ($\uparrow$) [a]	($\uparrow$) [b]
Protein-peptide binding affinity	Standard	Regression	Chemical FP	MAPc-8	70 %	$0.8\pm 0.1$
Protein-peptide binding affinity	Modified	Regression	Chemical FP	MAPc-20	80 %	$0.95\pm 0.03$
Cell penetration	Standard	Classification	Chemical FP	MAPc-8	60 %	$0.98\pm 0.04$
Cell penetration	Modified	Classification	Chemical FP	MAPc-12	60 %	$0.5\pm 0.2$
Antibacterial	Standard	Classification	Chemical FP	MAPc-8	60 %	$0.97\pm 0.02$
Antibacterial	Modified	Classification	Chemical FP	ECFP-12	50 %	$0.9\pm 0.1$
Antiviral	Standard	Classification	Sequence Alignment	MMSeqs2	80 %	$0.96\pm 0.05$
Antiviral	Modified	Classification	Chemical FP	MAPc-12	70 %	$0.6\pm 0.2$

*A) Best similarity functions for standard peptides *(Tables 2, S2, S4, S6, and S8). Surprisingly, fingerprint-based similarities performed better than sequence alignment for all standard datasets, except Antiviral where they are the second best choice. Among sequence alignments, MMSeqs2 with prior k-mer prefiltering is the best option. Interestingly, a prior study [14] had shown that MMSeqs2 with prefiltering can lead to lower sequence recall than without the prefiltering step or global alignment (Needleman-Wunsch). From our results, it seems that the worse recall from the alignment algorithm does not hinder its ability to properly partition the datasets. Overall, these results challenge established best practices for standard peptides that recommend using sequence alignments [14, 24, 43].

*B) Best similarity functions for modified peptides* (Tables 2, S3, S5, S7, and S9). The best options were also the chemical fingerprints, with three out of four datasets relying on the MAPc fingerprint and one on the ECFP fingerprint. This result is consistent with prior studies showing the superiority of MAPc over ECFP both for peptide chemical structure retrieval from databases [37, 44] and with their effect on model performance [22]. Chemical language model embeddings severely underperform with monotonicities under 0.5.

**Fig. 5:**
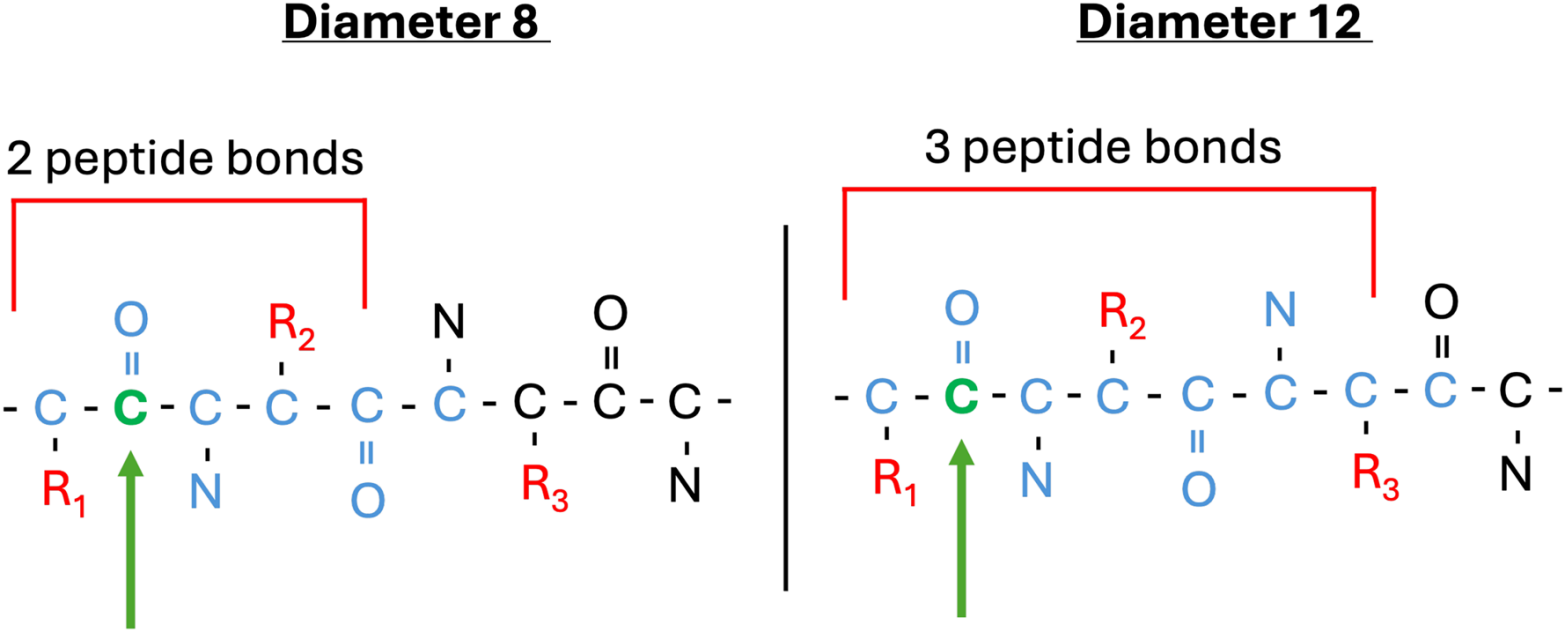
Illustration of peptide fingerprint environments with two different diameters, centered at the green atom. We only illustrate one half of the atomic environment for conciseness. $R_i$ refers to the side chain

*C) Fingerprint optimal diameter is determined by whether the peptides are standard or not*. Interestingly, MAPc with diameter 8 is the best fingerprint-based similarity function for all the standard datasets; whereas modified peptide datasets prefer larger diameters (12–20). This phenomenon could result from standard peptides not requiring as much resolution in the side chain composition (as there are only 20 fixed side chains). A diameter of 8, means that each atomic environment is comprised by all atoms at a maximum bond distance of 4 from the central atom, which limits the amount of side chain information available (Figure 5).

*D) Main recommendations*. The most versatile similarity function is Jaccard similarity with MAPc fingeprints, as it is the top choice in six of the eight scenarios here considered (Table 2). Attention has to be dedicated to the optimal fingeprint diameter, particularly with modified peptides, with the recommended options being diameters of at least 8. Additionally, MMSeqs2 with k-mer prefiltering and Jaccard (Tanimoto) similarity between ECFP fingerprints are robust alternatives for standard and for modified peptide datasets, respectively.

### Effect of peptide representation on model performance

We first considered the effect of choice of peptide representations in an interpolation scenario, where the training and testing peptides belong to the same class: standard or not. We isolated two different factors when designing our experiments: effect of the machine learning algorithm and effect of the representations.

**Fig. 6:**
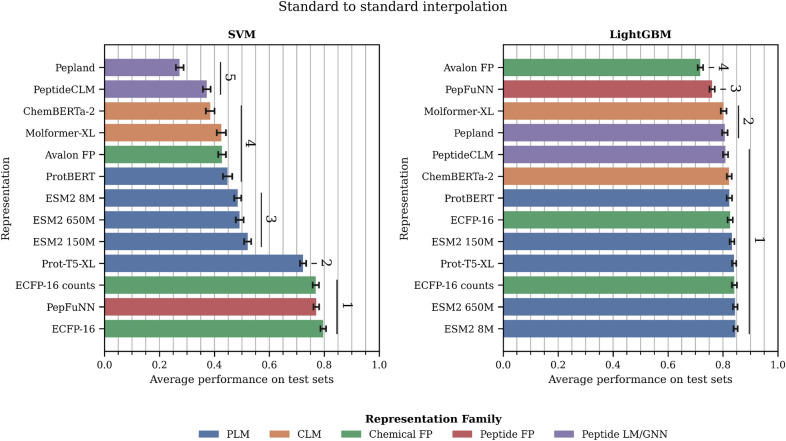
Evaluation of peptide representations in the standard to standard interpolation. Values are average performance (MCC for classification and SPCC for regression) across all training/testing splits at the different similarity thresholds of each dataset. Error bars: standard deviation. Vertical lines and numbers represent the significant rank each method belongs to. A significant rank is a group of models that are not significantly different ($p>0.004$) from the best model in the group

**Fig. 7:**
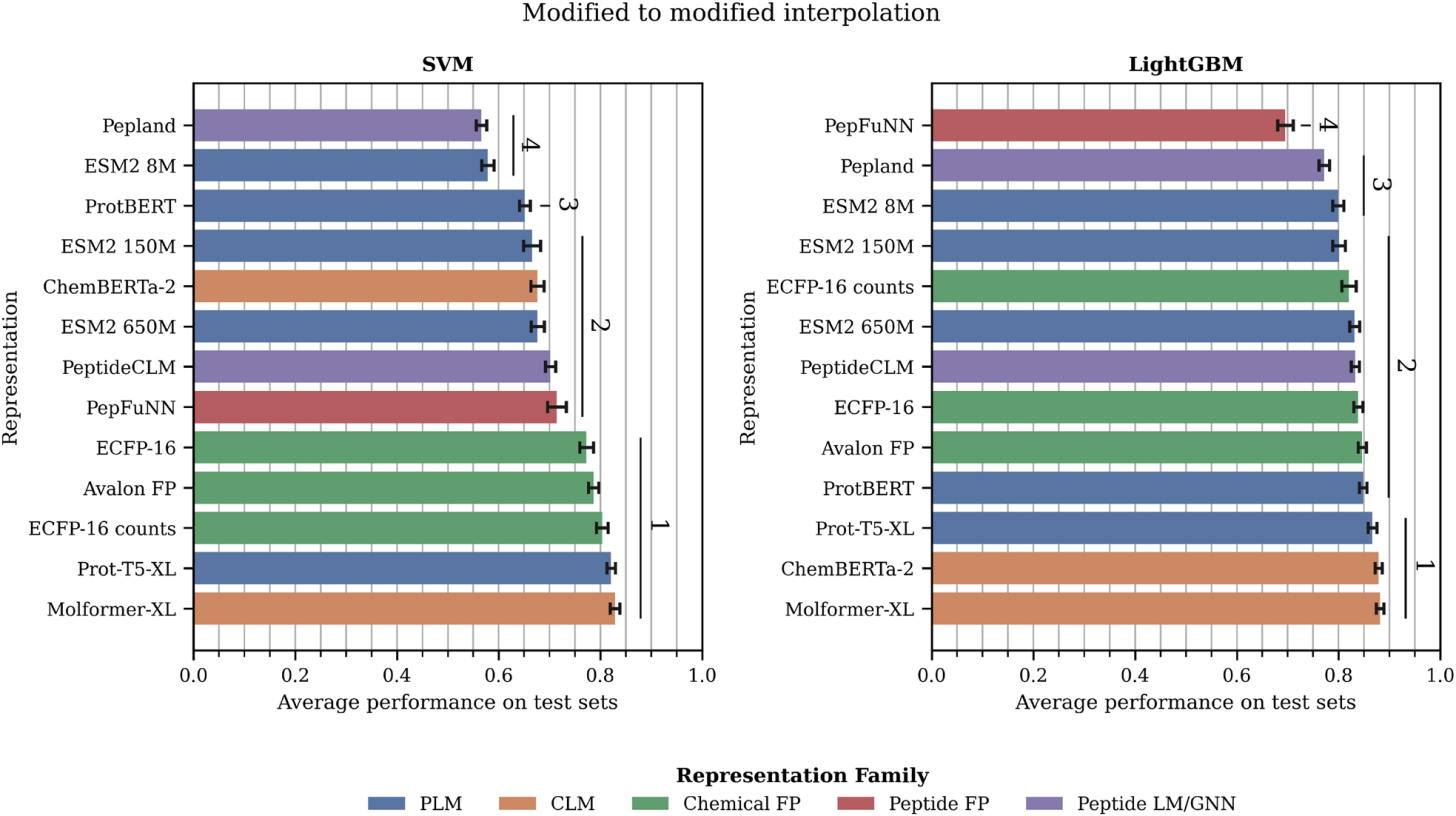
Evaluation of peptide representations in the modified to modified interpolation. Values are average performance (MCC for classification and SPCC for regression) across all training/testing splits at the different similarity thresholds of each dataset. Error bars: standard deviation. Vertical lines and numbers represent the significant rank each method belongs to. A significant rank is a group of models that are not significantly different ($p>0.004$) from the best model in the group

*A) Choice of machine learning algorithm* (Figures 6 and 7; Tables S11 and S12). We considered two lightweight ML algorithms as downstream models (SVM and LightGBM). LightGBM performs better on average than SVM. If we compare the top performance across representations, LightGBM shows better performance by 5% in both scenarios. Interestingly, the standard deviation across different representations varies with the downstream model: SVM has a greater standard deviation both for standard (SVM: 0.24; LightGBM: 0.12) and modified (SVM: 0.17; LightGBM: 0.13) datasets. Thus, the LightGBM models appear to be more robust to the representation used.

*B) Choice of representation*. The choice of peptide representation has a significant effect with both standard and modified peptide datasets, regardless of the downstream model (Kruskal-Wallis: $p<1\times 10^{-5}$ for all scenarios). The relative ranking of representations depends on the class of peptides.

*Standard to standard interpolation* (Figure 6 and Table S11). SVM clearly shows more differences between the representation methods, with molecular and peptide fingerprints ranking best (with the exception of Avalon FP), followed by the PLMs, then the CLMs, and lastly the Peptide LM/GNN. With LightGBM, the difference among representations sharply decreases, which is consistent with previous studies in standard datasets showing minimal difference among PLMs [24], and between PLMs and fingerprints [23].

*Modified to modified interpolation* (Figure 7 and Table S12). SVM again shows more discrimination between methods, but is less pronounced than in the standard to standard interpolation scenario. With SVM, all fingerprints (chemical and peptide) are ranked 1 alongside a CLM (Molformer-XL) and a PLM (Prot-T5-XL). With the LightGBM, the best options are both CLMs and Prot-T5-XL (PLM), followed by the fingerprints, several PLMs (ProtBERT, ESM2–150 M and ESM2–650 M), as well as PeptideCLM (Peptide LM). These results are surprising in how competitive PLMs are (most ranking 1 and 2) even with modified peptides where the modified residues are substituted by the ‘X’ amino acid. It is also surprising how low are the rankings of peptide-specific methods like PepFunn, Pepland, or PeptideCLM.

*C) General representation methods are better than peptide-specific ones in modified to modified interpolation*.

Unlike the findings for standard peptides, for the experiments with LightGBM on modified peptide datasets, the general chemical methods are clearly favoured over peptide-specific ones: CLMs (Molformer-XL and ChemBERTa-2) are both better than Peptide LM/GNNs (PeptideCLM and Pepland); and chemical fingerprints (ECFP-16, ECFP-16 counts, Avalon FP) are better than peptide fingerprints (PepFuNN). For standard datasets, however, this observation is not true, as all methods perform quite similarly.

*Limitations*. Our analyses are limited to an evaluation of these models as representation engines without finetuning. For example, on finetuning experiments, PeptideCLM has reported better performance on cell effective permeability prediction for modified peptides than ChemBERTa-2 [29]. We release our data and partitions to allow other researchers to benchmark alternative modeling approaches.^2^

*D) Combination of representations*. Finally, we also tried the combination of the top representations from the LightGBM experiments, by concatenating the individual representations. The statistical analysis of the results showed no significant benefit of the combination compared to using single representations (Figure S11).

### Model performance in standard to modified extrapolation

Standard peptides are cheaper and easier to synthesize than modified ones. Thus, models that are able to extrapolate from standard to modified peptides would allow researchers to leverage the results from cheaper experiments to reduce the number of more costly experiments. However, no previous study has been conducted to examine whether current representation techniques are able to perform this generalization step. As our datasets are paired, with standard and modified peptides for the same task, we can evaluate the ability of the models to extrapolate between peptide classes (standard to modified) by comparing training the models on the standard datasets and evaluating them against the modified peptide datasets. In this case, training and test data are already different from each other (standard to modified) so we do not need to examine generalization at multiple similarity thresholds. Thus, the test dataset will be the complete modified dataset. We perform 25 independent runs with random training subsets (sampling without replacement 80% of the training data) of the standard datasets, to isolate the noise related to the training data composition.

**Fig. 8:**
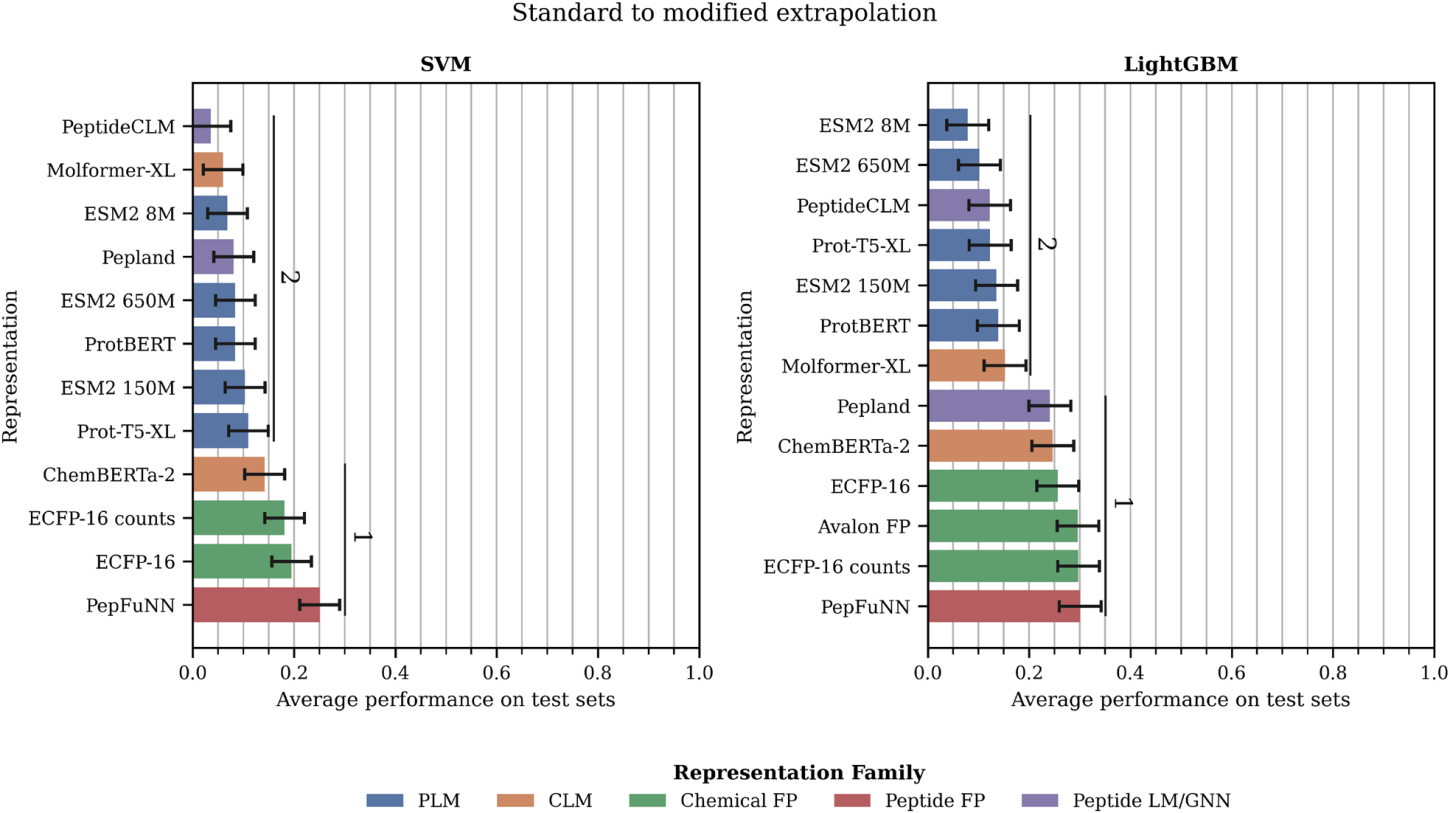
Evaluation of peptide representations in the standard to modified extrapolation scenario. Values are average performance (MCC for classification and SPCC for regression) across all 25 independent runs. Error bars: standard deviation. Vertical lines and numbers represent the significant rank each method belongs to. A significant rank is a group of models that are not significantly different ($p>0.05$) from the best model in the group

*A) Choice of machine learning algorithm* (Figure 8 and Table S13). LightGBM performs better on average than the SVM, with top performance increased by 7%. In this case, there is no difference in the standard deviation across different representations with the downstream model (SVM: 0.18 and LightGBM: 0.19).

*B) Choice of representation* (Figure 8 and Table S13). Molecular and peptide fingerprints are the best choices along with one of the CLMs (ChemBERTa-2) and the Peptide LM (PeptideCLM). It is particularly surprising the case of the peptide fingerprint (PepFuNN) as it the worst and second to worst in the modified and standard interpolation scenarios, respectively. This observation might indicate that this fingerprint loses some resolution when comparing peptides from the same class, but keeps enough information as to be easily transferred from one class to the other. Interestingly, the PLMs which where performing competitively in the two interpolation scenarios, here they show significantly worse results. This happens regardless of whether the modified residues are substituted by ‘X’ (ESM2–8 M: $0.08\pm 0.04$; Prot-T5-XL: $0.12\pm 0.04$) or their natural analog (ESM2–8 M: $0.09\pm 0.04$; Prot-T5-XL: $0.11\pm 0.04$)

*C) Discussion on the difference between methods within the same family*. It is worth considering the difference between the two CLMs: ChemBERTa-2 (rank 1) and Molformer-XL (rank 2); and between the two Peptide LM/GNN: PeptideCLM (rank 1) and Pepland (rank 2).

*C.1) Difference between CLMs.* Both CLMs have Transformers architectures and have been trained with a Masked Language Modeling (MLM) objective, where they try to predict tokens within a SMILES string that have been previously randomly masked. In this work, Molformer-XL refers to the publicly available version of the model which was trained on $\approx 100\times 10^6$ molecules sourced from PubChem and ZINC (1:10). On the other hand, ChemBERTa-2 is trained on $\approx 77\times 10^6$ molecules from only PubChem. ZINC is a decoy database that is enriched on smaller molecules. Another difference between both approaches is that the sequence length in Molformer-XL is restricted to 202 tokens (a token would correspond to an atom or another gramatical rule like branching or cycles); whereas ChemBERTa-2 is restricted to 512. Both factors suggest that Molformer’s training data is more biased towards smaller organic compounds, whereas ChemBERTa-2 has been exposed to a greater proportion of peptides (and other macromolecules) during pre-training, explaining its better performance.

*C.2) Difference between Peptide LM/GNN.* Pepland and PeptideCLM architectures are different (GNN and Transformer, respectively), but they both have atomic resolution and, in principle, they have access to the same information. Regarding their pretraining, they both try to work around the scarcity of peptide data by pretraining their models in two stages: 1) a general stage with more diverse molecules to learn general rules about organic chemistry and SMILES notation which relies on $\approx 10^6$ molecules for training; and 2) a specialisation phase on modified peptides. The two models differ in the composition and size of this second dataset. Pepland used a smaller dataset of experimentally validated cyclic peptides with some ability to penetrate cell membranes collected in CycPeptMPDB [45] ($\approx 8,000$ molecules) and PeptideCLM used a larger, computationally generated dataset of cyclic modified peptides with $\approx 10\times 10^6$ samples. Pepland’s pre-training dataset seems to be quite biased towards cyclic cell-penetrating peptides, but it provides more meaningful representations for real bioactive compounds. PeptideCLM’s dataset, however, is similarly restricted to cyclic peptides, and although larger, it may carry unwanted biases related to the computational pipeline used to generate the peptides, which may affect its representations of real molecules.

## Conclusions

This study provides a systematic evaluation of peptide representations and models for modeling standard and modified peptides. Our findings indicate that molecular similarity functions for guiding dataset partitioning are better suited for standard peptide datasets than sequence alignments, challenging the conventional practice.

Regarding the choice of downstream model, simpler models like Support Vector Machines showed more sensitivity towards the representation technique; whereas more complex models like LightGBM were more robust and achieved better overall performance. Regarding the choice of representations, chemical fingerprints are the best choice for standard to standard interpolation alongside Protein Language Models; whereas Chemical Language Models are a better choice for modified to modified peptide interpolation.

Crucially, when considering the more challenging scenario of extrapolation from standard to modified peptides, chemical fingerprints are again the best option available. Therefore, we would recommend their usage for peptide machine learning predictive modelling, in all cases, except where most of the training and inference data is modified; in which case Chemical Language Models, particularly ChemBERTa-2, will be better suited.

Finally, to facilitate ongoing research and innovation in peptide representation learning, we publicly release the benchmark datasets along with partitioning schemes to guide the evaluation and development of molecular representation techniques for peptides.

## Supplementary information

Supplementary file 1.

## Data Availability

Data and code are made publicly available in the associated Github repository: https://github.com/IBM/PeptideGeneralizationBenchmarks

## Footnotes

1 https://github.com/zhangruochi/pepland

2 https://github.com/IBM/PeptideGeneralizationBenchmarks
