# Supplementary material for: How to build machine learning models able to extrapolate from standard to modified peptides
Source: J Cheminform. 2025 Nov 27;17:185. doi: 10.1186/s13321-025-01115-z (PMC12751563; doi:10.1186/s13321-025-01115-z)
Supplement: Supplementary file 1 [file 13321_2025_1115_MOESM1_ESM.pdf]

Supplementary Information for paper: How to build machine learning models able to extrapolate from standard to modified peptides

Raúl Fernández-Díaz
Conway Institute for Biomolecular and Biomedical Research
School of Medicine,
The SFI Center for Research Training on Genomics Data Science
University College Dublin, Dublin, Ireland

IBM Research, Dublin, Ireland
raul.fernandezdiaz@ucdconnect.ie

Rodrigo Ochoa
Novo Nordisk A/S, Måløv, Denmark

Thanh Lam Hoang, Vanessa Lopez
IBM Research, Dublin, Ireland

Denis Shields
Conway Institute for Biomolecular and Biomedical Research
School of Medicine,
The SFI Center for Research Training on Genomics Data Science
University College Dublin, Dublin, Ireland
denis.shields@ucd.ie

A Benchmark dataset construction

A.1 Protein-peptide binding affinity

The two protein-peptide binding affinity datasets were collected from [1]. Entries with modified peptides were removed from the standard dataset and entries with standard peptides were removed from the modified dataset. In total, 805 peptides were removed from the standard dataset and 5, from the modified.

Figure S1 displays the distribution of physicochemical characteristics of the benchmark datasets.

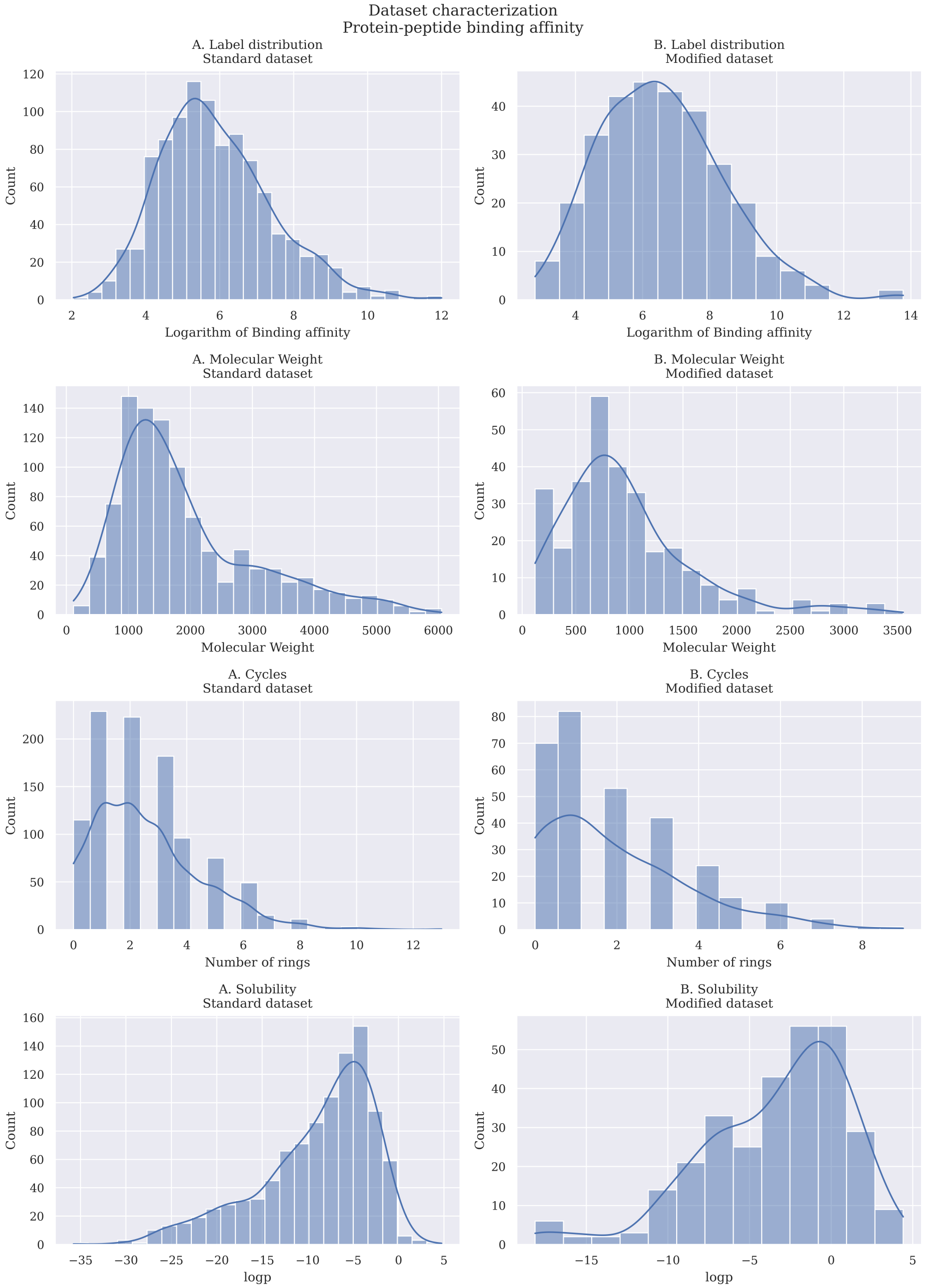

Figure S1: Protein-peptide binding affinity benchmark datasets.

A.2 Cell penetration

The two cell penetration datasets were collected from [1]. The standard dataset only contained standard peptides, so no further removal was necessary.

The standard dataset was a classification task and the modified, a regression task. Further, the label distribution for this dataset shows a high peak at exactly -10 (see Figure S2), which corresponds to the detection limit of the assays used to build this dataset [2].

For these two reasons, we decided to redefine modified dataset as a classification task where the negative class was defined as all peptides with -10 PAMPA permeability and the positives as those between -6.5 and -4.5. This range for the positives was selected to provide peptides closer to the mean of the distribution. We undersampled the positive class, to obtain a balanced dataset. In the end the dataset was comprised of 480 peptides.

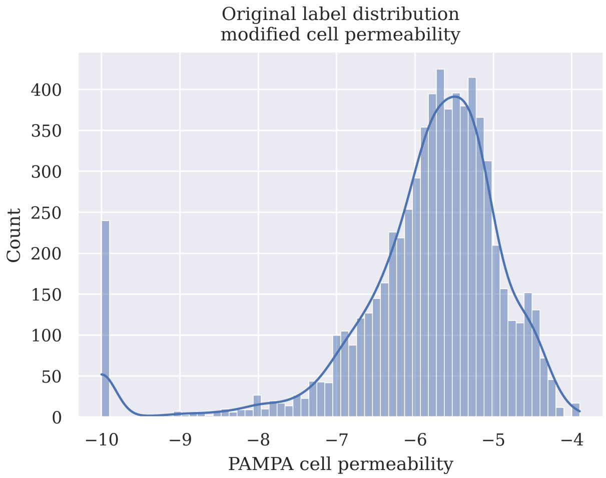

Figure S2: Label distribution for original modified cell penetration dataset

Figure S3 displays the distribution of physicochemical characteristics of the final benchmark datasets.

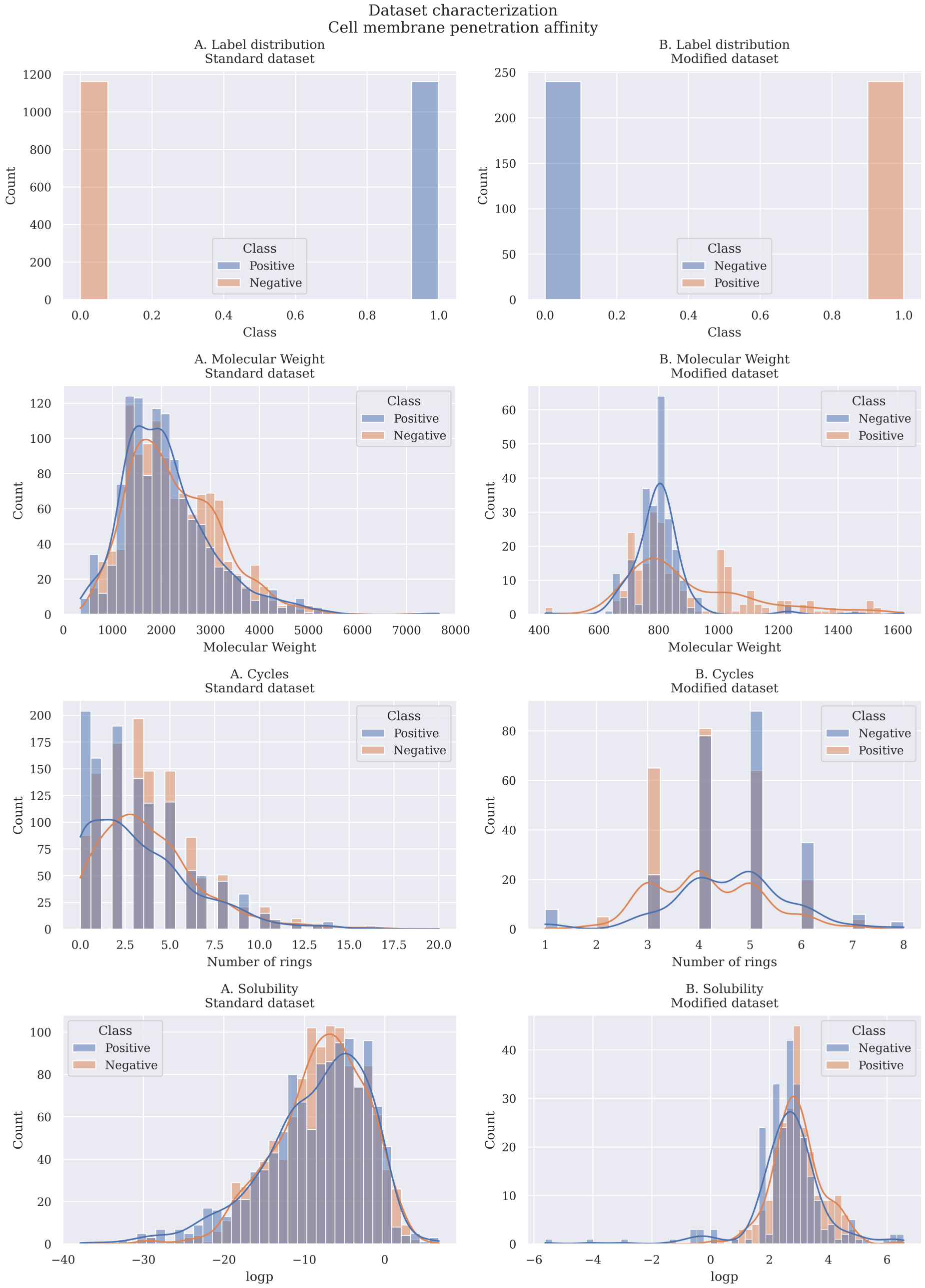

Figure S3: Cell penetration benchmark datasets.

A.3 Antibacterial

The standard antibacterial dataset was collected from [3]. Duplicated peptides and peptides with more than 50 residues where removed, which affected 3,370 entries.

The modified antibacetrial dataset was collected from [4]. The negative class was defined with the same strategy as [3], as other modified peptides with different bioactivities (i.e., antiviral, antifungal, antiparasitic).

Figure S4 describes the physicochemical characteristics of the final benchmark datasets.

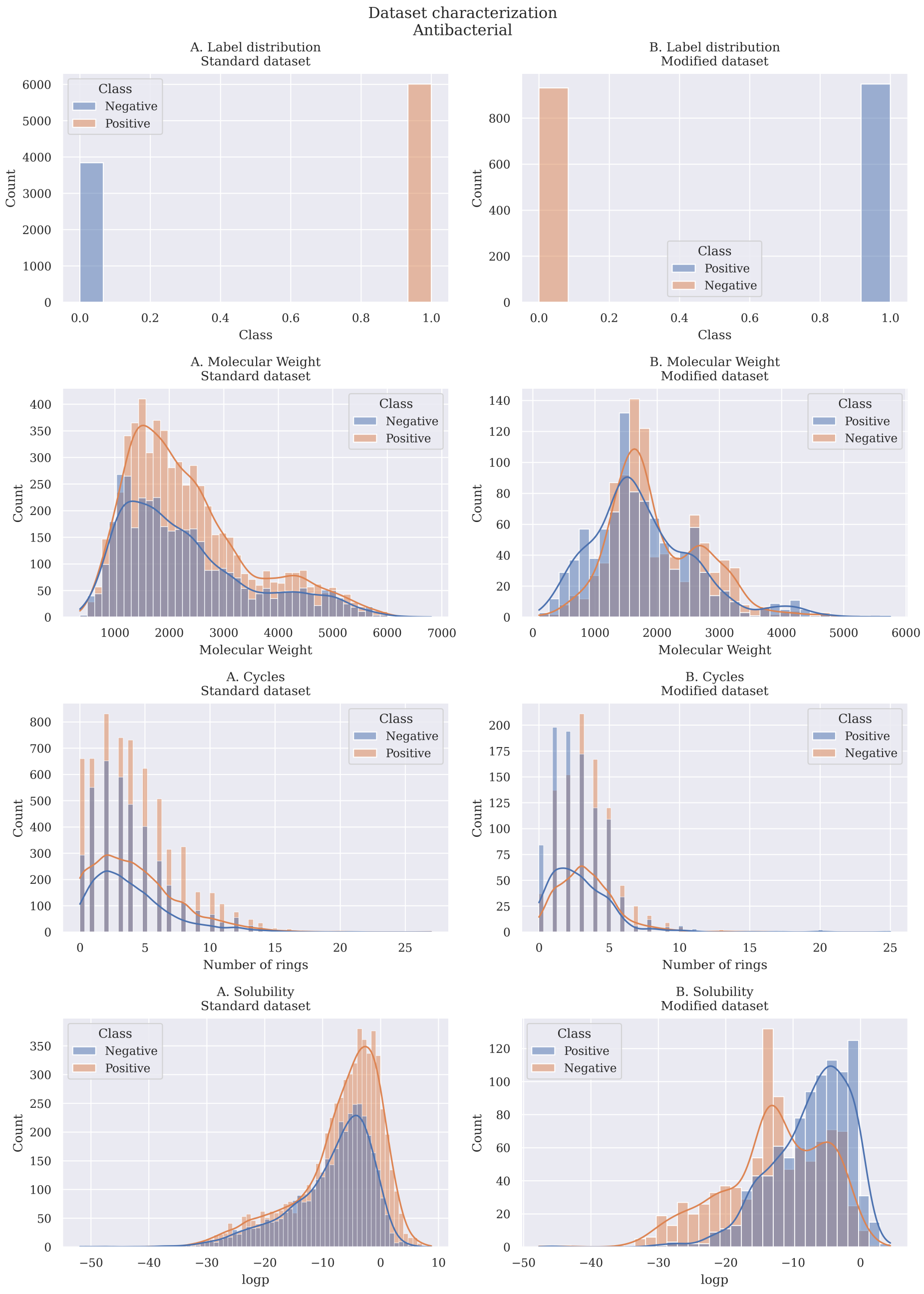

Figure S4: Antibacterial benchmark datasets.

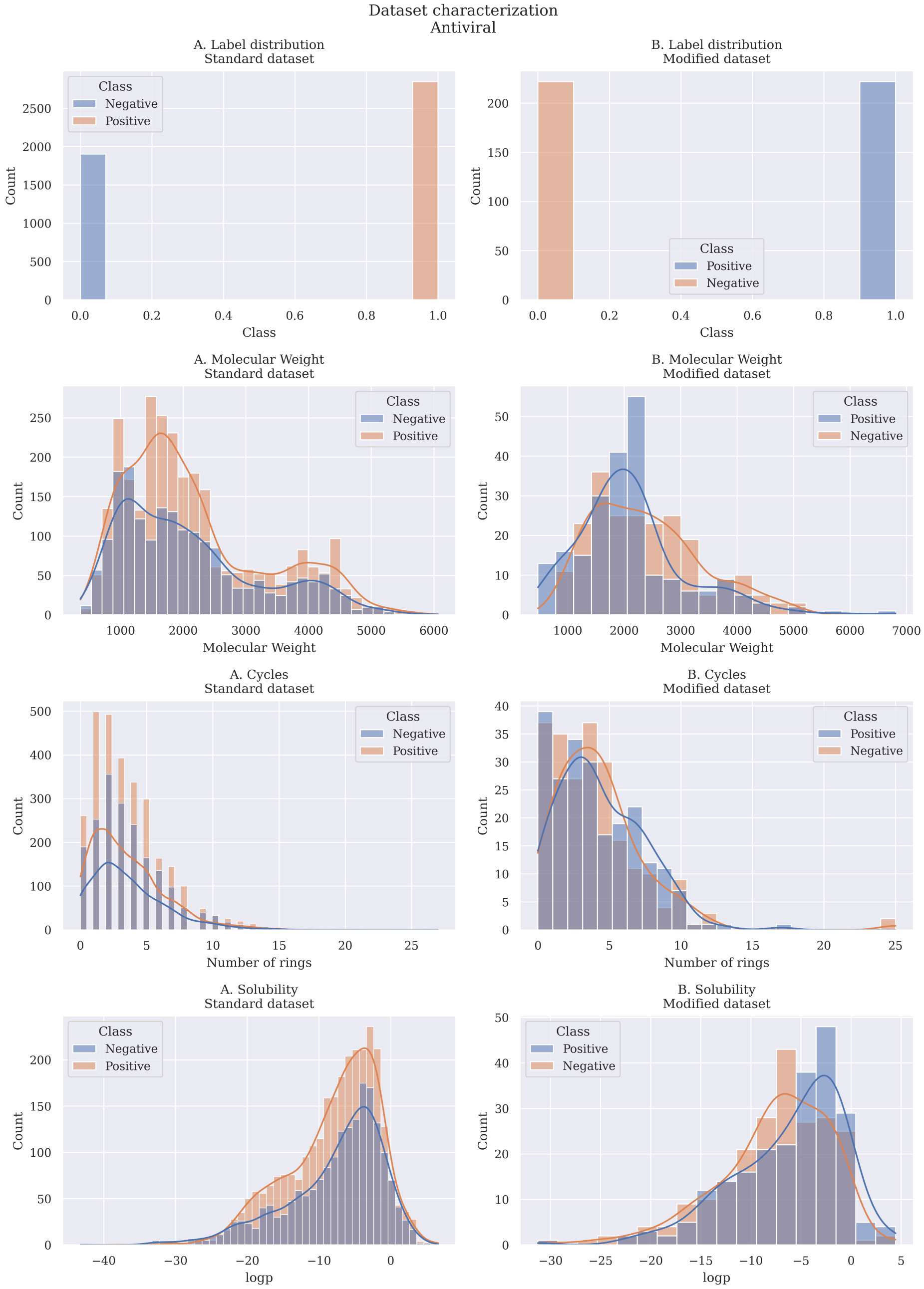

Figure S5: Antiviral benchmark datasets.

A.4 Antiviral

The standard antiviral dataset was collected from [3]. Duplicated peptides and peptides with more than 50 residues where removed, which affected 160 entries.

The modified antiviral dataset was collected from [4]. The negative class was defined with the same strategy as [3], as other modified peptides with different bioactivities (i.e., antibacterial, antifungal, antiparasitic).

Figure S5 describes the physicochemical characteristics of the final benchmark datasets.

B Similarity functions

B.1 MMSeqs2 configuration

The MMSeqs2 [5] configuration used for computating sequence alignment between standard peptides, followed the default settings from Hestia-GOOD [6] (v.1.0.3), using longest as denominator for sequence identity [3].

B.2 Needleman-Wunsch

The global alignment with Needleman-Wunsch algorithm was performed using the default configuration in the EMBOSS `needleall` implementation [7], it is as follows:

```
needleall -aformat pair -gapopen 10 -gapextend 0.5 -endopen 10

-endextend 0.5 -datafile EBLOSSUM62
```

This code is called for each peptide pair, and is run in parallel using the corresponding function in the Hestia-GOOD package.

B.3 Molecular fingerprints

The calculation of similarity between the molecular fingerprints ECFP and MAPc is performed using the corresponding functions from the Hestia-GOOD version `0.1.0` package, which relies on RDKit [8] for the ECFP fingerprints and on the MAPc package [1].

C Machine learning modelling

C.1 Hyperparameter optimization

Table S1 contains the hyperparameter space considered for the hyperparameter optimization through Bayesian Optimization with Optuna [9]. The Bayesian optimization was performed with default settings for 200 steps with 10 parallel threads and early stopping with a patience of 20.

Table S1: Hyperparameter search space for each learning algorithm.

**Model**	**Trials**	**Hyperparameter search space**			
		**Name**	**Type**	**Range**	**Log-scale**
SVM	200	`C`	float	1×10^{-3} - 10^{3}	Yes
		`kernel`	categorical	linear, poly, rbf, sigmoid	NA
		`degree` (only kernel poly)	integer	2 - 5	No
		`coef0` (only with poly or sigmoid)	float	10^{-8} - 1	Yes
		`epsilon` (only regression)	float	10^{-5} - 1	Yes
LightGBM	200	`n_estimators` (number of estimators)	integer	10 - 500	No
		`min_split_gain`	float	10^{-10} - 10^{-3}	Yes
		`reg_alpha` (α regularization)	float	10^{-10} - 10^{-3}	Yes
		`learning_rate`	float	10^{-7} - 10^{-1}	Yes

C.2 Peptide representations

All molecular language models were applied using their `huggingface` public weights. They are as follows:

- ESM2-8M: `facebook/esm2_t6_8M_UR50D`
- ESM2-150M: `facebook/esm2_t30_150M_UR50D`
- Prot-T5-XL: `Rostlab/prot_t5_xl_half_uniref50-enc`
- ChemBERTa-2 77M MLM: `DeepChem/ChemBERTa-77M-MLM`

[1] `https://github.com/markusorsi/mapchiral`

- Molformer-XL: `ibm/MoLFormer-XL-both-10pct`
- PeptideCLM: `aaronfeller/PeptideCLM-23M-all`

For PeptideCLM, the code for the tokenizer was obtained from its public repository[2]. For Pepland, the code for extracting the representations and model weights were also obtained from its public repository[3].

The ECFP and Avalon fingerprints were calculated using the corresponding function in RDKit, with radius 8 and number of bits 2,048. The choice of radius 8 was made after a non-exhaustive exploration through a subset of the datasets. Performance of these fingerprints could reasonably be enhanced by including radius and number of bits as additional hyperparameters.

Representation	Antibacterial	Antiviral	Cell penetration binding affinity	Protein-peptide	Average	Significant rank
Prot-T5-XL - X	0.78±0.01	0.91±0.01	0.81±0.02	0.78±0.01	0.82±0.01	1
Prot-T5-XL - *	0.83±0.01	0.65±0.02	0.77±0.03	0.78±0.02	0.75±0.01	1
ESM2-8M - *	0.82±0.01	0.61±0.02	0.48±0.02	0.76±0.02	0.67±0.01	2
ESM2 8M - X	0.73±0.01	0.61±0.02	0.52±0.03	0.50±0.02	0.58±0.01	3

Table S2: Comparison of sequence reconstruction methods for modified peptides. X: modified residues are substituted by 'X'; '*': they are substituted by their natural analog.

[2] `https://github.com/AaronFeller/PeptideCLM`
[3] `https://github.com/zhangruochi/pepland`

D Search for optimal similarity function

Figure S6 shows the results for the search for optimal similarity function and it highlights, for each dataset, the function selected. Tables S2 - S9, contain the metrics for each of the similarity functions.

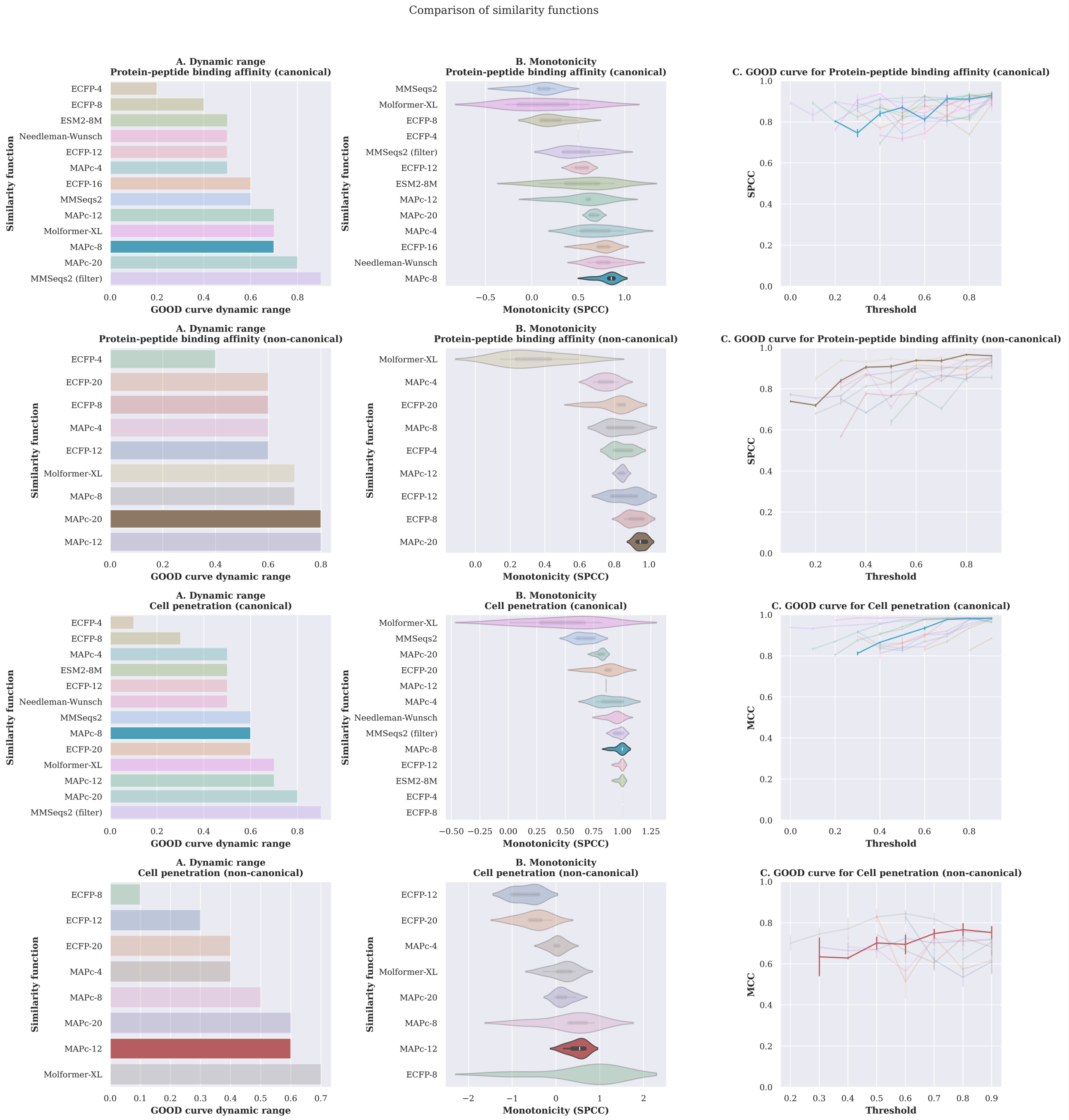

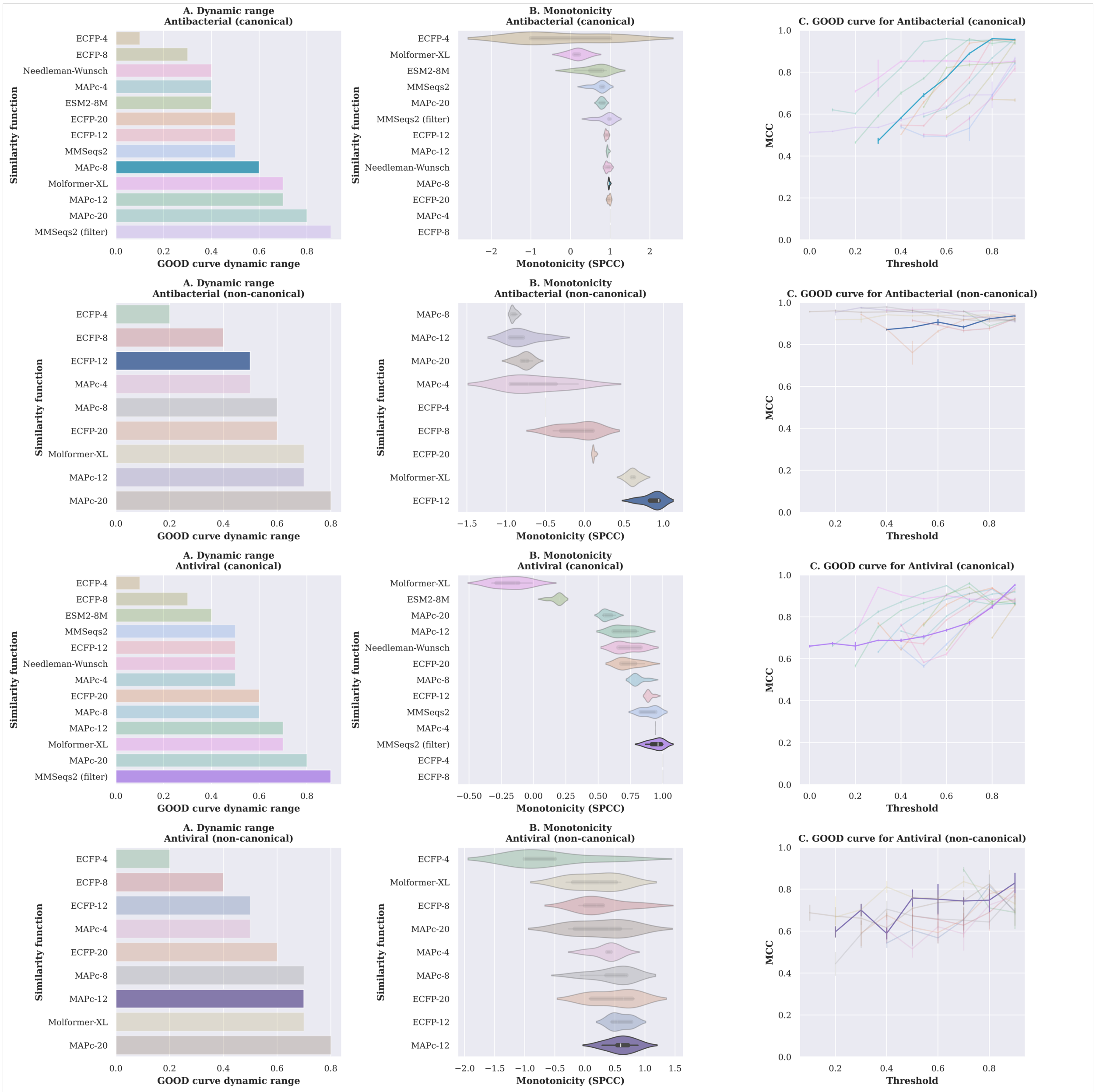

Figure S6: Results per similarity function per dataset

Table S3: Similarity function evaluation for protein-peptide binding affinity (standard) dataset. Green: Optimal similarity function.

Similarity	Monotonicity	Dynamic range
MAPc-20	0.67±0.05	80
MMSeqs2 (filter)	0.5±0.2	80
MAPc-8	0.8±0.1	70
MAPc-12	0.6±0.2	70
Molformer-XL	0.1±0.4	70
ECFP-16	0.7±0.1	60
MMSeqs2	0.0 ±0.2	60
ECFP-12	0.53±0.07	50
ESM2-8M	0.5±0.3	50
MAPc-4	0.7±0.2	50
Needleman-Wunsch	0.8±0.1	50
ECFP-8	0.2±0.2	40
ECFP-4	0.50±0.00	20

Table S4: Similarity function evaluation for protein-peptide binding affinity (modified) dataset. Green: Optimal similarity function.

Similarity	Monotonicity	Dynamic range
MAPc-20	0.95±0.03	80
MAPc-12	0.84±0.02	80
MAPc-8	0.83±0.08	70
Molformer-XL	0.3±0.2	70
ECFP-12	0.87±0.08	60
ECFP-20	0.8±0.1	60
ECFP-8	0.91±0.05	60
MAPc-4	0.75±0.06	60
ECFP-4	0.84±0.05	40

Table S5: Similarity function evaluation for cell penetration (standard). Green: Optimal similarity function.

Similarity	Monotonicity	Dynamic range
MMSeqs2 (filter)	0.97±0.05	80
MAPc-20	0.81±0.04	80
MAPc-12	0.86±0.00	70
Molformer-XL	0.5±0.3	70
MAPc-8	0.98±0.05	60
ECFP-20	0.9±0.1	60
MMSeqs2	0.66±0.08	60
ECFP-12	0.99±0.03	50
ESM2-8M	0.99±0.03	50
MAPc-4	0.9±0.1	50
Needleman-Wunsch	0.93±0.06	50
ECFP-8	1.00±0.00	30
ECFP-4	1.00±0.00	10

Table S6: Similarity function evaluation for cell penetration (modified) dataset. Green: Optimal similarity function.

**Similarity**	**Monotonicity**	**Dynamic range**
Molformer-XL	0.1±0.3	70
MAPc-12	0.5±0.2	60
MAPc-20	0.2±0.2	60
MAPc-8	0.3±0.6	50
ECFP-20	-0.5±0.3	40
MAPc-4	0.0±0.2	40
ECFP-12	-0.7±0.3	30
ECFP-8	0.6±0.9	10

Table S7: Similarity function evaluation for antibacterial (standard) dataset. Green: Optimal similarity function

**Similarity**	**Monotonicity**	**Dynamic range**
MMSeqs2 (filter)	0.9±0.2	80
MAPc-20	0.79±0.06	80
MAPc-12	0.94±0.02	70
Molformer-XL	0.2±0.2	70
MAPc-8	0.97±0.02	60
ECFP-12	0.91±0.03	50
ECFP-20	0.98±0.03	50
MMSeqs2	0.73±0.2	50
ESM2-8M	0.6±0.3	40
MAPc-4	1.00±0.00	40
Needleman-Wunsch	0.94±0.05	40
ECFP-8	1.00±0.00	30
ECFP-4	0±1	10

Table S8: Similarity function evaluation for antibacterial (modified) dataset. Green: Optimal similarity function.

**Similarity**	**Monotonicity**	**Dynamic range**
MAPc-20	-0.77±0.09	80
MAPc-12	-0.8±0.2	70
Molformer-XL	0.62±0.07	70
ECFP-20	0.11±0.02	60
MAPc-8	-0.91±0.03	60
ECFP-12	0.9±0.1	50
MAPc-4	-0.6±0.4	50
ECFP-8	-0.1±0.2	40
ECFP-4	-0.50±0.00	20

Table S9: Similarity function evaluation for antiviral (standard) dataset. Green: Optimal similarity function.

**Similarity**	**Monotonicity**	**Dynamic range**
MMSeqs2 (filter)	0.96±0.05	80
MAPc-20	0.57±0.04	80
MAPc-12	0.71±0.09	70
Molformer-XL	-0.2±0.1	70
MAPc-8	0.81±0.05	60
ECFP-20	0.74±0.08	60
ECFP-12	0.90±0.03	50
MAPc-4	0.94±0.00	50
MMSeqs2	0.90±0.06	50
Needleman-Wunsch	0.73±0.09	50
ESM2-8M	0.18±0.04	40
ECFP-8	1.00±0.00	30
ECFP-4	1.00±0.00	10

Table S10: Similarity function evaluation for antiviral (modified) dataset. Green: Optimal similarity function.

Similarity	Monotonicity	Dynamic range
MAPc-20	0.3±0.5	80
MAPc-12	0.6±0.2	70
MAPc-8	0.5±0.3	70
Molformer-XL	0.2±0.4	70
ECFP-20	0.5±0.4	60
ECFP-12	0.6±0.2	50
MAPc-4	0.4±0.2	50
ECFP-8	0.3±0.4	40
ECFP-4	-0.6±0.7	20

E Analysis of different representations

This section contains the results, per dataset, of the main representations.

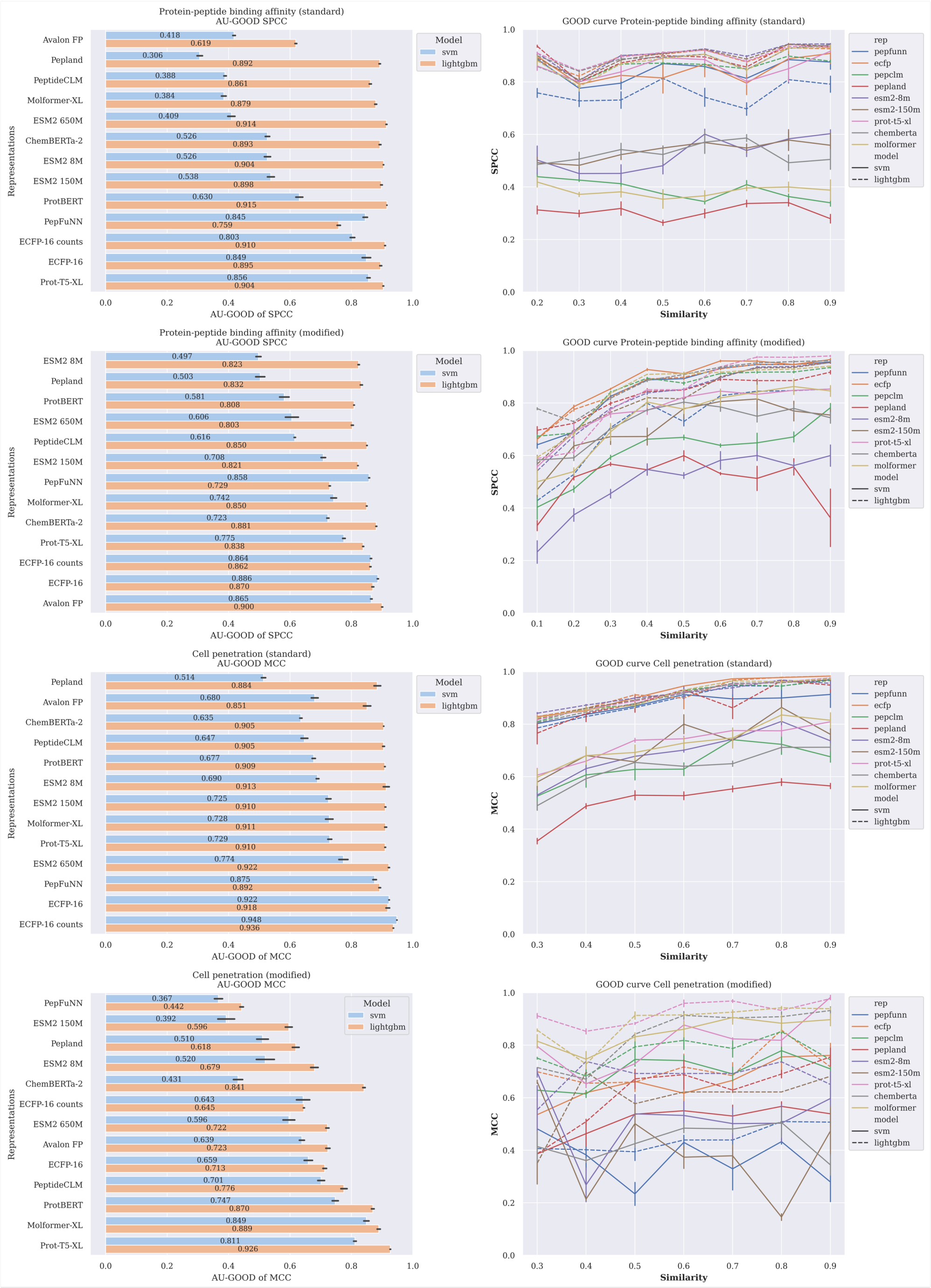

Figure S7: Results for all experiments both with SVM and LightGBM.

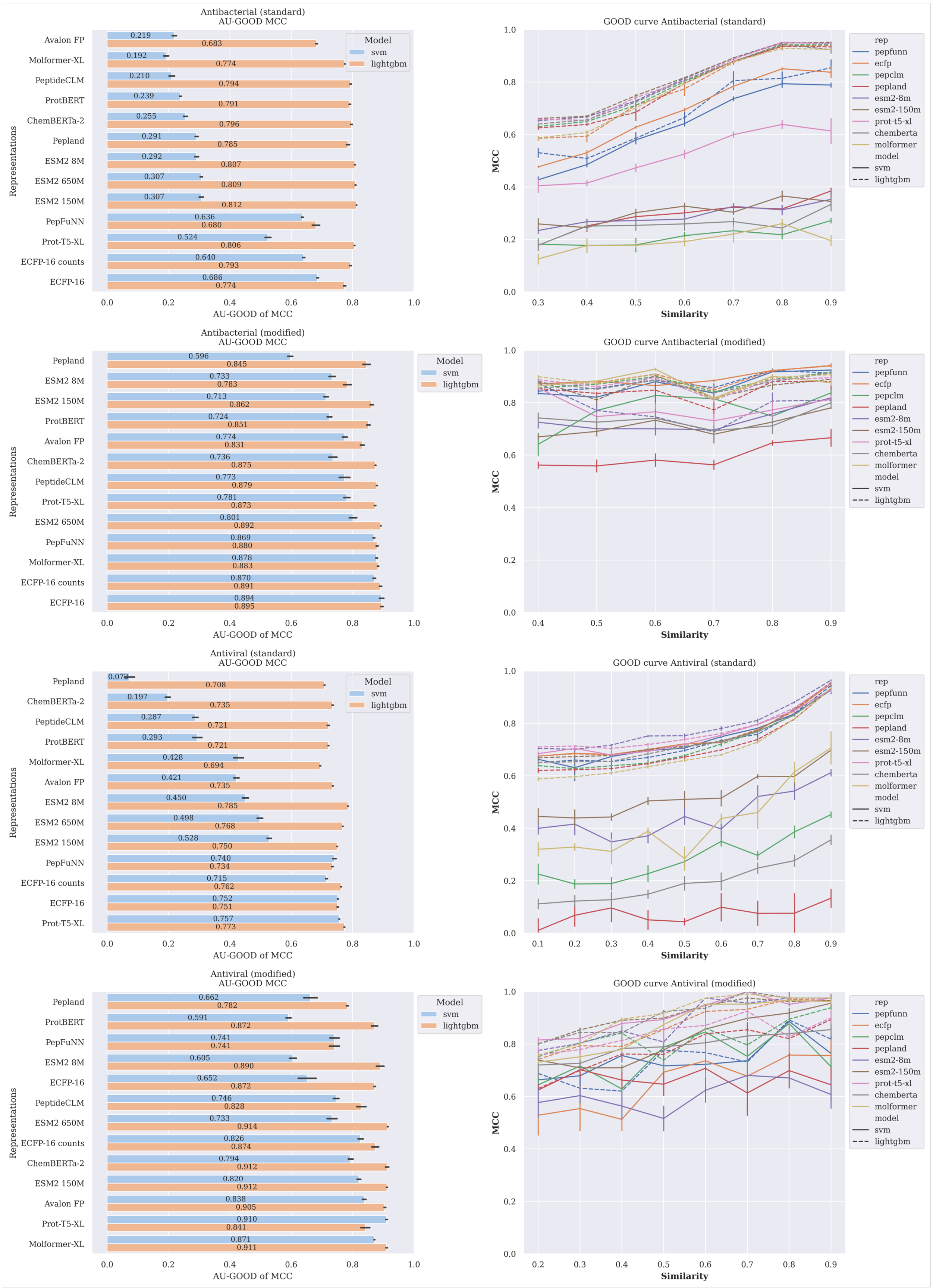

Figure S8: Results for all experiments both with SVM and LightGBM.

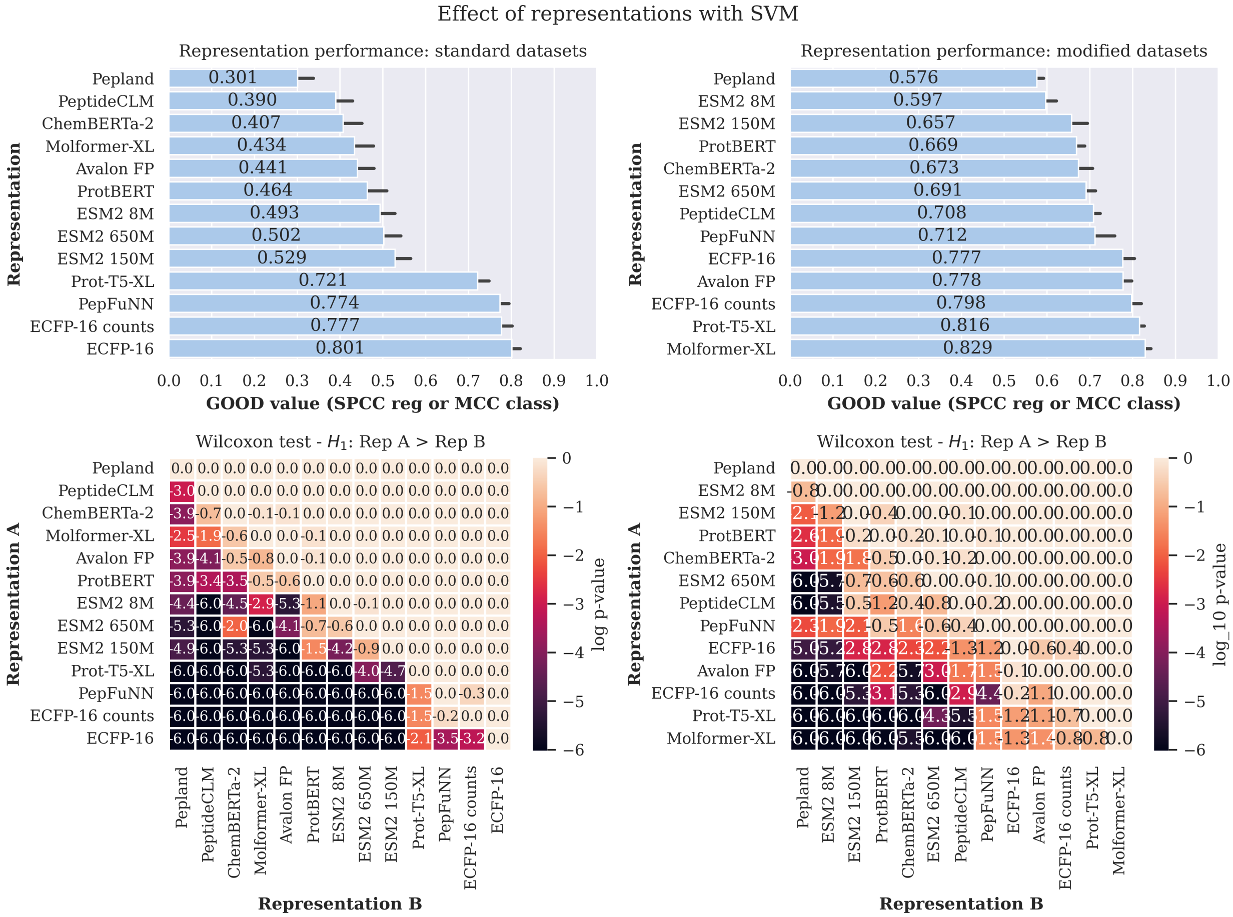

Figure S9: Comparison of representations using SVM. Heatmaps correspond to the log p-values of the pairwise Wilcoxon test.

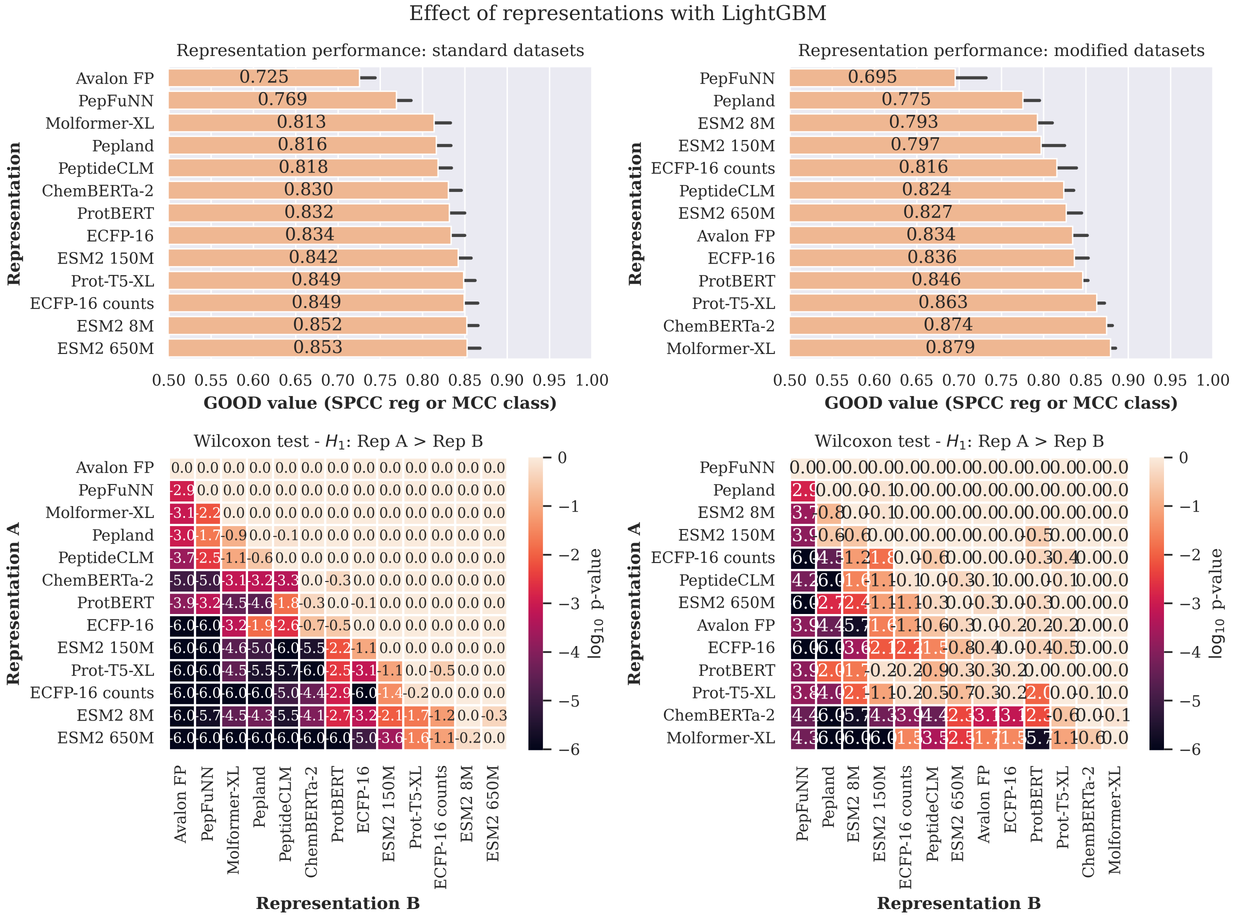

Figure S10: Combination of representations using LightGBM. Heatmaps correspond to the log p-values of the pairwise Wilcoxon test.

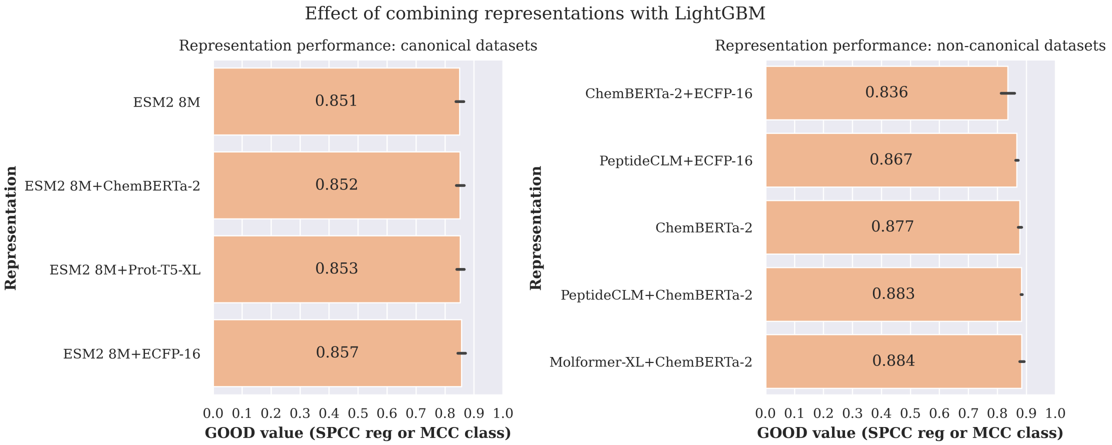

Figure S11: Results for the experiments with LightGBM combining multiple representations

SVM

Representation	Protein-peptide binding affinity (standard)	Antibacterial (standard)	Antiviral (standard)	Cell penetration (standard)	**Average**	Significant rank
ECFP-16	0.69±0.02	0.74±0.01	0.92±0.01	0.85±0.01	0.80±0.01	1
PepFuNN	0.64±0.02	0.73±0.01	0.88±0.01	0.84±0.01	0.77±0.01	1
ECFP-16 counts	0.64±0.02	0.71±0.01	0.95±0.01	0.80±0.01	0.77±0.01	1
Prot-T5-XL	0.52±0.02	0.75±0.01	0.85±0.01	0.84±0.01	0.75±0.01	1
ESM2 150M	0.31±0.01	0.51±0.01	0.72±0.02	0.52±0.01	0.51±0.01	2
ESM2 650M	0.31±0.01	0.49±0.01	0.77±0.01	0.41±0.01	0.49±0.01	2
ESM2 8M	0.29±0.01	0.45±0.01	0.68±0.01	0.52±0.01	0.48±0.01	2
ProtBERT	0.24±0.01	0.29±0.02	0.68±0.02	0.63±0.01	0.45±0.02	2
Molformer-XL	0.19±0.01	0.41±0.02	0.73±0.02	0.38±0.01	0.43±0.02	3
Avalon FP	0.22±0.01	0.40±0.02	0.68±0.02	0.42±0.00	0.43±0.01	3
ChemBERTa-2	0.25±0.01	0.19±0.01	0.64±0.01	0.53±0.01	0.38±0.02	4
PeptideCLM	0.21±0.01	0.28±0.01	0.65±0.02	0.39±0.01	0.37±0.01	4
Pepland	0.29±0.01	0.07±0.01	0.51±0.01	0.31±0.01	0.27±0.01	5

LightGBM

Representation	Protein-peptide binding affinity (standard)	Antibacterial (standard)	Antiviral (standard)	Cell penetration (standard)	**Average**	Significant rank
ESM2 8M	0.81±0.02	0.78±0.01	0.91±0.01	0.90±0.01	0.85±0.01	1
ESM2 650M	0.81±0.02	0.76±0.01	0.92±0.01	0.91±0.00	0.84±0.01	1
ECFP-16 counts	0.79±0.02	0.75±0.01	0.94±0.01	0.91±0.01	0.84±0.01	1
Prot-T5-XL	0.81±0.02	0.77±0.01	0.91±0.01	0.90±0.00	0.84±0.01	1
ESM2 150M	0.81±0.02	0.74±0.01	0.91±0.01	0.90±0.01	0.83±0.01	1
ECFP-16	0.77±0.02	0.74±0.01	0.92±0.01	0.90±0.01	0.83±0.01	1
ChemBERTa-2	0.80±0.02	0.73±0.01	0.90±0.01	0.89±0.01	0.82±0.01	1
ProtBERT	0.79±0.02	0.71±0.01	0.91±0.01	0.92±0.01	0.82±0.01	1
PeptideCLM	0.79±0.02	0.71±0.01	0.90±0.01	0.86±0.00	0.81±0.01	1
Pepland	0.78±0.02	0.70±0.01	0.88±0.01	0.89±0.01	0.81±0.01	2
Molformer-XL	0.77±0.02	0.68±0.02	0.91±0.01	0.88±0.01	0.80±0.01	2
PepFuNN	0.68±0.02	0.73±0.01	0.89±0.01	0.76±0.01	0.76±0.01	3
Avalon FP	0.68±0.02	0.73±0.01	0.85±0.02	0.62±0.01	0.72±0.01	4

Table S11: **Standard to standard interpolation.** Average performance of the representations per dataset. Errors correspond to the standard error of the mean. Significant rank: tier that the model occupies based on the pairwise statistical comparisons. Colors: chemical fingerprints (green); peptide fingerprint (red); CLMs (orange); PLMs (blue); peptide LM/GNN (pink). Rows are ordered by column Average.

SVM

Representation	Protein-peptide binding affinity (modified)	Antibacterial (modified)	Antiviral (modified)	Cell penetration (modified)	**Average**	Significant rank
Molformer-XL	0.88±0.01	0.87±0.02	0.85±0.01	0.74±0.02	0.83±0.01	1
Prot-T5-XL	0.78±0.01	0.91±0.01	0.81±0.02	0.78±0.01	0.82±0.01	1
ECFP-16 counts	0.87±0.01	0.83±0.02	0.64±0.02	0.86±0.01	0.80±0.01	1
Avalon FP	0.77±0.01	0.84±0.01	0.64±0.01	0.86±0.02	0.79±0.01	1
ECFP-16	0.89±0.01	0.65±0.03	0.66±0.02	0.89±0.01	0.77±0.01	1
PepFuNN	0.87±0.01	0.74±0.02	0.37±0.02	0.86±0.02	0.71±0.02	2
PeptideCLM	0.77±0.02	0.75±0.02	0.70±0.01	0.62±0.02	0.70±0.01	2
ESM2 650M	0.80±0.01	0.73±0.02	0.60±0.02	0.61±0.03	0.68±0.01	2
ChemBERTa-2	0.74±0.01	0.79±0.01	0.43±0.02	0.72±0.01	0.68±0.01	2
ESM2 150M	0.71±0.01	0.82±0.02	0.39±0.03	0.71±0.02	0.67±0.02	2
ProtBERT	0.72±0.01	0.59±0.02	0.75±0.02	0.58±0.02	0.65±0.01	3
ESM2 8M	0.73±0.01	0.61±0.02	0.52±0.03	0.50±0.02	0.58±0.01	4
Pepland	0.60±0.01	0.66±0.02	0.51±0.02	0.50±0.02	0.57±0.01	4

LightGBM

Representation	Protein-peptide binding affinity (modified)	Antibacterial (modified)	Antiviral (modified)	Cell penetration (modified)	**Average**	Significant rank
Molformer-XL	0.88±0.01	0.91±0.01	0.89±0.01	0.85±0.02	0.88±0.01	1
ChemBERTa-2	0.87±0.00	0.91±0.01	0.84±0.02	0.88±0.01	0.88±0.01	1
Prot-T5-XL	0.87±0.01	0.84±0.02	0.93±0.01	0.84±0.02	0.87±0.01	1
Avalon FP	0.83±0.01	0.90±0.01	0.72±0.01	0.90±0.01	0.85±0.01	2
ProtBERT	0.85±0.01	0.87±0.02	0.87±0.01	0.81±0.02	0.85±0.01	2
ECFP-16	0.90±0.01	0.87±0.01	0.71±0.01	0.87±0.01	0.84±0.01	2
ESM2 650M	0.89±0.00	0.91±0.01	0.72±0.01	0.80±0.02	0.83±0.01	2
PeptideCLM	0.88±0.00	0.83±0.02	0.78±0.01	0.85±0.01	0.83±0.01	2
ECFP-16 counts	0.89±0.01	0.87±0.01	0.65±0.04	0.86±0.02	0.82±0.01	2
ESM2 150M	0.86±0.01	0.91±0.01	0.60±0.02	0.82±0.02	0.80±0.01	2
ESM2 8M	0.78±0.01	0.89±0.02	0.68±0.02	0.82±0.02	0.80±0.01	3
Pepland	0.85±0.01	0.78±0.01	0.62±0.02	0.83±0.01	0.77±0.01	3
PepFuNN	0.88±0.01	0.74±0.02	0.44±0.01	0.73±0.02	0.70±0.02	4

Table S12: **Modified to modified interpolation.** Average performance of the representations per dataset. Errors correspond to the standard error of the mean. Significant rank: tier that the model occupies based on the pairwise statistical comparisons. Colors: chemical fingerprints (green); peptide fingerprint (red); CLMs (orange); PLMs (blue); peptide LM/GNN (pink). Rows are ordered by column Average.

SVM

Representation	Protein-peptide binding affinity	Antibacterial	Antiviral	Cell penetration	**Average**	Significant rank
PepFuNN	0.32±0.06	0.49±0.03	-0.04±0.04	0.23±0.03	0.25±0.04	1
ECFP-16	0.25±0.06	0.54±0.03	-0.08±0.04	0.08±0.03	0.19±0.04	1
ECFP-16 counts	0.27±0.06	0.51±0.03	-0.09±0.04	0.03±0.03	0.18±0.04	1
ChemBERTa-2	0.02±0.06	0.14±0.03	0.03±0.04	0.37±0.03	0.14±0.04	1
Prot-T5-XL	0.08±0.06	0.01±0.03	0.01±0.04	0.34±0.03	0.11±0.04	2
ESM2 150M	0.08±0.06	-0.00±0.03	0.02±0.04	0.31±0.03	0.10±0.04	2
ESM2 650M	-0.01±0.06	0.09±0.03	0.11±0.04	0.14±0.03	0.08±0.04	2
ProtBERT	0.02±0.06	0.02±0.03	0.05±0.04	0.24±0.03	0.08±0.04	2
Pepland	0.20±0.06	-0.06±0.03	0.07±0.04	0.12±0.03	0.08±0.04	2
ESM2 8M	0.08±0.06	0.01±0.03	-0.03±0.04	0.21±0.03	0.07±0.04	2
Molformer-XL	0.07±0.06	0.01±0.03	0.06±0.04	0.11±0.03	0.06±0.04	2
PeptideCLM	-0.01±0.06	0.03±0.03	-0.14±0.04	0.26±0.03	0.04±0.04	2

LightGBM

Representation	Protein-peptide binding affinity	Antibacterial	Antiviral	Cell penetration	**Average**	Significant rank
PepFuNN	0.40±0.02	0.47±0.03	-0.03±0.05	0.36±0.02	0.30±0.04	1
Avalon FP	0.33±0.02	0.40±0.03	0.04±0.05	0.41±0.02	0.30±0.04	1
ECFP-16 counts	0.30±0.02	0.54±0.03	-0.02±0.05	0.36±0.02	0.30±0.04	1
ECFP-16	0.32±0.02	0.47±0.03	-0.12±0.05	0.36±0.02	0.26±0.04	1
ChemBERTa-2	0.31±0.02	0.39±0.03	0.02±0.05	0.26±0.02	0.25±0.04	1
Pepland	0.19±0.02	0.31±0.03	0.12±0.05	0.34±0.02	0.24±0.04	1
Molformer-XL	0.32±0.02	0.06±0.03	-0.14±0.05	0.36±0.02	0.15±0.04	2
ESM2 150M	0.04±0.02	-0.04±0.03	0.17±0.05	0.36±0.02	0.14±0.04	2
ProtBERT	0.01±0.02	0.14±0.03	0.13±0.05	0.27±0.02	0.14±0.04	2
Prot-T5-XL	0.03±0.02	0.04±0.03	0.07±0.05	0.35±0.02	0.12±0.04	2
PeptideCLM	0.21±0.02	0.06±0.03	-0.19±0.05	0.41±0.02	0.12±0.04	2
ESM2 650M	0.00±0.02	0.06±0.03	0.09±0.05	0.25±0.02	0.10±0.04	2
ESM2 8M	-0.04±0.02	0.00±0.03	0.02±0.05	0.33±0.02	0.08±0.04	2

Table S13: **Standard to modified extrapolation.** Average performance of the representations per dataset. Errors correspond to the standard error of the mean. Significant rank: tier that the model occupies based on the pairwise statistical comparisons. Colors: chemical fingerprints (green); peptide fingerprint (red); CLMs (orange); PLMs (blue); peptide LM/GNN (pink). Rows are ordered by column Average.

References

[1] Ruochi Zhang, Haoran Wu, Yuting Xiu, Kewei Li, Ningning Chen, Yu Wang, Yan Wang, Xin Gao, and Fengfeng Zhou. Pepland: a large-scale pre-trained peptide representation model for a comprehensive landscape of both canonical and non-canonical amino acids. *arXiv preprint arXiv:2311.04419*, 2023.

[2] Jianan Li, Keisuke Yanagisawa, Masatake Sugita, Takuya Fujie, Masahito Ohue, and Yutaka Akiyama. Cycpeptm-pdb: A comprehensive database of membrane permeability of cyclic peptides. *Journal of Chemical Information and Modeling*, 63(7):2240–2250, 2023.

[3] Raúl Fernández-Díaz, Rodrigo Cossio-Pérez, Clement Agoni, Hoang Thanh Lam, Vanessa Lopez, and Denis C Shields. Autopeptideml: a study on how to build more trustworthy peptide bioactivity predictors. *Bioinformatics*, 40(9):btae555, 2024.

[4] Micael da Silva Pirazoli Gonzalez, Caio Cheohen, Bruce Veiga Andriolo, and Manuela Leal da Silva. Development of a database of peptides with potential for pharmacological intervention in human pathogen molecular targets. *Latin American Data in Science*, 3(1):16–21, 2023.

[5] Martin Steinegger and Johannes Söding. Mmseqs2 enables sensitive protein sequence searching for the analysis of massive data sets. *Nature biotechnology*, 35(11):1026–1028, 2017.

[6] Raúl Fernández-Díaz, Hoang Thanh, Vanessa Lopez, and Denis C Shields. A new framework for evaluating model out-of-distribution generalisation for the biochemical domain. In *The Thirteenth International Conference on Learning Representations*, 2025.

[7] Peter Rice, Ian Longden, and Alan Bleasby. Emboss: the european molecular biology open software suite. *Trends in genetics*, 16(6):276–277, 2000.

[8] Greg Landrum. Rdkit documentation. *Release*, 1(1-79):4, 2013.

[9] Takuya Akiba, Shotaro Sano, Toshihiko Yanase, Takeru Ohta, and Masanori Koyama. Optuna: A next-generation hyperparameter optimization framework. In *Proceedings of the 25th ACM SIGKDD international conference on knowledge discovery & data mining*, pages 2623–2631, 2019.
